# Randomized Trial of Lower-Dose Roxadustat Efficacy and Safety in Non–Dialysis-Dependent CKD-Associated Anemia

**DOI:** 10.1016/j.ekir.2025.01.027

**Published:** 2025-01-27

**Authors:** Ping Li, Xuefeng Sun, Li Zhang, Hongli Lin, Niansong Wang, Yuehong Li, Sumei Zhao, Ping Fu, Hong Cheng, Zhiyong Guo, Wanhong Lu, Yani He, Fengmin Shao, Qiang He, Yiqing Wu, Cuihua Huang, Shuting Pan, Guangyan Cai, Xiangmei Chen, FGCL-4592-858 Working Group, Xiangmei Chen, Guangyan Cai, Ping Li, Xuefeng Sun, Li Zhang, Hongli Lin, Niansong Wang, Yuehong Li, Sumei Zhao, Ping Fu, Hong Cheng, Zhiyong Guo, Wanhong Lu, Yani He, Fengmin Shao, Qiang He, Shiren Sun, Wei Liang, Hongtao Yang, Zhaohui Ni, Qiongqiong Yang, Wenge Li, Aihua Zhang, Guojuan Zhang, Gengru Jiang, Bo Lin, Yanning Zhang, Wenhu Liu, Yonghui Mao, Jinsheng Xu, Weiping Liu, Song Wang, Xiaodong Zhang, Jurong Yang, Hongwei Jiang, Yiqing Wu, Cuihua Huang, Shuting Pan

**Affiliations:** 1 Department of Nephrology, First Medical Center of Chinese PLA General Hospital, State Key Laboratory of Kidney Diseases, National Clinical Research Center for Kidney Diseases, Beijing Key Laboratory of Medical Devices and Integrated Traditional Chinese and Western Drug Development for Severe Kidney Diseases, Beijing Key Laboratory of Digital Intelligent TCM for the Prevention and Treatment of Pan-vascular Diseases, Key Disciplines of National Administration of Traditional Chinese Medicine (zyyzdxk-2023310), Beijing, China; 2 Department of Nephrology, The First Affiliated Hospital of Dalian Medical University, Dalian, Liaoning, China; 3 Department of Nephrology, Shanghai Sixth People’s Hospital Affiliated to Shanghai Jiao Tong University, Shanghai, China; 4 Department of Nephrology, Beijing Tsinghua Changgung Hospital, Clinical Medicine of Tsinghua University, Beijing, China; 5 Department of Nephrology, Beijing Chao-Yang Hospital, Capital Medical University, Beijing, China; 6 Department of Nephrology, West China Hospital, Sichuan University, Chengdu, China; 7 Department of Nephrology, Beijing Anzhen Hospital, Capital Medical University, Beijing, China; 8 Department of Nephrology, The First Affiliated Hospital of Naval Medical University, Changhai Hospital, Shanghai, China; 9 Department of Nephrology, The First Affiliated Hospital of Xi’an Jiao Tong University, Xi’an, China; 10 Department of Nephrology, Army Medical Center of Chinese People’s Liberation Army, Chongqing, China; 11 Department of Nephrology, Henan Provincial People’s Hospital, Zhengzhou, China; 12 Department of Nephrology, The First Affiliated Hospital of Zhejiang Chinese Medical University, Zhejiang Provincial Hospital of Traditional Chinese Medicine, Hangzhou, China; 13 Medical Affairs and Clinical Biometrics Department, FibroGen (China) Medical Technology Development Company Ltd., Beijing, China

**Keywords:** anemia, chronic kidney disease, efficacy, hemoglobin variability, roxadustat

## Abstract

**Introduction:**

Different starting doses of roxadustat are used for treating anemia in chronic kidney disease (CKD). We tested the noninferiority of weight-based lower starting dose compared with standard starting dose roxadustat for anemia in stage 3 to 5 CKD without dialysis.

**Methods:**

Patients were randomized (1:1) and stratified by CKD stage to receive weight-based standard (< 60 kg: 70 mg 3 times per week [TIW]; ≥ 60 kg: 100 mg TIW) or 1-step-lower (< 60 kg: 50 mg TIW; ≥ 60 kg: 70 mg TIW) roxadustat starting dose for 16 weeks. The primary endpoint was mean hemoglobin change from baseline over weeks 12 to 16. The secondary endpoints included the proportion achieving hemoglobin 100 to 120 g/l, hemoglobin variability, and rescue therapy.

**Results:**

Overall, 254 patients were randomized. The mean (SD) baseline hemoglobin was 89.88 (6.90) g/l. Most patients had stage 4 (39.0%) or stage 5 (40.2%) CKD. Mean hemoglobin increased from baseline at weeks 12 to 16 (lower: 21.57 g/l; standard: 26.35 g/l), but noninferiority was not met. A comparable proportion achieved hemoglobin of 100 to 120 g/l (lower: 46.0%; standard: 47.2%). The hemoglobin increase was comparable in CKD stage 3 to 4, but less with the lower dose in CKD stage 5 (17.28 vs. 26.71 g/l). The lower dose exhibited a lower hemoglobin rate of change (lower: 2.917; standard: 3.376) and less drug exposure. Drug-related adverse event rates were comparable.

**Conclusion:**

The proportion of patients who achieved the hemoglobin target was comparable between the doses. The lower starting dose had less hemoglobin fluctuation and is recommended for stage 3 to 4 CKD.

## Graphical abstract

**Figure:**
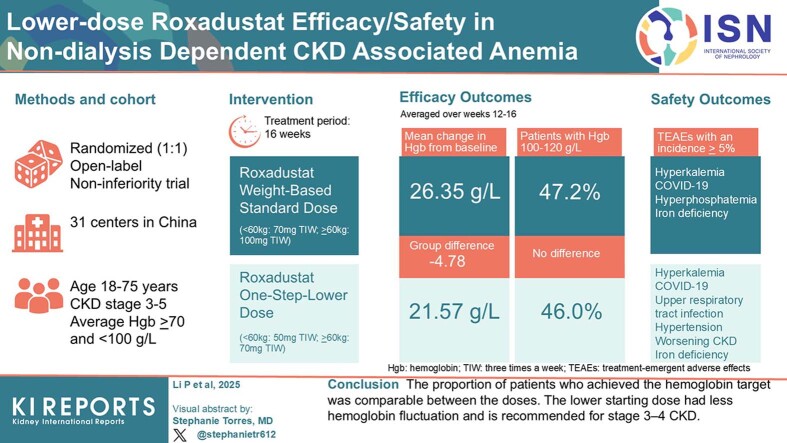

Treating anemia in CKD with conventional therapies is challenging because of hyporesponsiveness of erythropoiesis-stimulating agent (ESA) and the low proportion of patients meeting the target hemoglobin range.^1^^,^^2^ In addition, anemia in CKD is often undertreated worldwide,^3^, ^4^, ^5^ and in China, only 44.9% of patients with CKD with anemia receive conventional anemia medication.^6^

Hypoxia-inducible factor (HIF) prolyl-hydroxylase inhibitors (PHIs) are relatively novel alternative treatment options for the clinical management of anemia in CKD. HIF-PHIs mimic the hypoxia-driven endogenous expression of erythropoietin. The European Renal Association suggests the clinical use of HIF-PHI in non–dialysis-dependent (NDD) or peritoneal dialysis patients who prefer oral treatment; have difficulty starting or receiving ESAs; have issues receiving iron therapy or when increased iron availability is desired; are hyporesponsive to or intolerant of, ESAs; or have chronic inflammatory states (C-reactive protein ≥ 3 mg/l).^7^

Roxadustat is the first-in-class oral HIF-PHI, and its efficacy and safety have been well-documented in patients with anemia in dialysis-dependent and NDD CKD in phase 3 trials.^8^, ^9^, ^10^, ^11^, ^12^ Roxadustat is approved for the treatment of anemia in CKD in several countries^11^^,^^13^ with varying starting doses in clinical practice. Although some small-scale studies in NDD patients from the US^14^ and Japan^15^^,^^16^ have shown that lower fixed starting doses (50 mg) of roxadustat facilitate slower achievement of the hemoglobin response than fixed 70 mg/100 mg doses, no studies have assessed the efficacy and safety of roxadustat comparing weight-based lower starting doses and the standard, which are approved starting doses in China. In addition, studies of patients on dialysis have shown that both low and high hemoglobin concentrations are associated with increased cardiovascular events and death, and the proportion of time that hemoglobin remains above or below the target threshold may be associated with adverse outcomes.^17^ Therefore, hemoglobin fluctuation at treatment initiation and its short-term outcomes should also be evaluated.

This dose-optimization study was conducted to identify a more effective, safer, and more economic roxadustat starting dose. We hypothesized that the efficacy of weight-based lower starting dose of roxadustat would be noninferior compared with the standard starting dose in Chinese patients with anemia in stage 3 to 5 NDD CKD.

## Methods

### Study Design

This randomized (1:1), controlled, open-label, noninferiority trial, conducted from December 2021 to April 2023, compared the efficacy and safety of weight-based standard approved (< 60 kg: 70 mg; ≥ 60 kg: 100 mg) or 1-step-lower (< 60 kg: 50 mg; ≥ 60 kg: 70 mg) starting doses of roxadustat^18^ in patients with anemia in stage 3 to 5 NDD CKD at 31 centers in China (clinical trial registration ChiCTR2100045359). The treatment period was 16 weeks, and the posttreatment follow-up period was 4 weeks. All patients provided written informed consent. The study was approved by the ethics committee of Chinese PLA General Hospital (approval no. S202-523-05) and other sites and was performed in accordance with the Declaration of Helsinki, as revised in 2013. The detailed study design has been published previously.^19^

### Patient Enrollment

The key inclusion criteria were the following: (i) age 18 to 75 years; (ii) stage 3 to 5 CKD with an estimated glomerular filtration rate < 60 ml/min per 1.73 m^2^ (CKD Epidemiology Collaboration equation)^20^ and not expecting to undergo renal replacement therapy within 6 months; (iii) no treatment with ESAs within the previous 4 weeks, or with roxadustat (or any other HIF-PHI) within the previous 12 weeks (and thus considered ESA-naïve and HIF-PHI-naïve); (iv) The mean hemoglobin level at screening and baseline is ≥ 70 g/l and < 100 g/l; and (v) body weight ≥ 40 kg. The full inclusion and exclusion criteria are provided in Supplementary Table S1.

### Randomization

The patients were randomized 1:1 to standard or lower starting dose of roxadustat with stratification by CKD stage (3–5). The planned sample size was 250 (125 in each group). The random numbers were expanded by about 50% to generate a total of 360 random numbers (180 in each group). A block randomization design (block size of 2; generated by the randomization statistician using SAS 9.4 software) and a pregenerated subject randomization table were used. The randomization table was imported into the Clinflash IRT system, and the random allocation sequence was implemented using Interactive Response Technologies. Patients were randomized to either the lower starting dose or standard starting dose group and were enrolled and assigned to the interventions by the investigators.

### Treatment

The patients were administered either the weight-based standard starting dose (< 60 kg: 70 mg TIW; ≥ 60 kg: 100 mg TIW) or the 1-step-lower starting dose (< 60 kg: 50 mg TIW; ≥ 60 kg: 70 mg TIW). Dose adjustments were made every 4 weeks based on the current hemoglobin concentration and change in hemoglobin over the previous 4 weeks (Supplementary Methods and Supplementary Table S2). Oral supplemental iron could be administered at any time according to clinical practice. I.V. iron was not allowed unless administered as rescue therapy (Supplementary Methods). The treatment duration was 16 weeks.

### Efficacy Assessment

The primary efficacy endpoint was the mean change in hemoglobin from baseline averaged over weeks 12 to 16. The secondary efficacy endpoints were as follows: (i) the proportion of patients who achieved a hemoglobin concentration of 100 to 120 g/l averaged over weeks 12 to 16; (ii) hemoglobin variability based on the proportion of time that hemoglobin was outside the range of 100 to 130 g/l from baseline to weeks 4, 8, and 16; the summed absolute rate of change in hemoglobin from baseline to weeks 4, 8, and 16; and the change in the hemoglobin linear regression slope from baseline to week 4, weeks 4 to 8, and weeks 8 to 16; and (iii) the proportion of patients who received rescue therapy (composite of blood transfusion, ESA, and i.v. iron) and time to rescue therapy from the date of the first dose (Supplementary Methods).

### Exploratory Endpoints Assessment

The time to first achieving a hemoglobin response (increase by ≥ 10 g/l from baseline) and the cumulative proportion of patients with a hemoglobin response from baseline at any time up to and including week 16, as well as the number of dose adjustments during the treatment period, were assessed.

### Safety Assessment

The numbers (%) of patients with treatment-emergent adverse events (TEAEs), drug-related TEAEs, treatment-emergent serious adverse events, and adverse events of special interest were assessed (defined in the Supplementary Methods). TEAEs were defined as any new condition or worsening of an existing condition after the first roxadustat dose and within 28 days after the last dose.

### Sample Size Determination

To provide a power of at least 85% to test the noninferiority of the weight-based lower dose to the standard dose in the primary endpoint with a noninferiority margin of −5 g/l (lower dose − standard dose), a common SD of < 12 g/l, and a loss to follow-up rate of 30% at a 2-sided significance of 0.05, we estimated that the enrollment of 250 patients would be sufficient. Noninferiority was established if the lower boundary of the 2-sided 95% confidence interval (CI) for the difference in hemoglobin change from baseline was greater than the noninferiority margin of −5 g/l. The noninferiority margin was chosen to preserve at least 70% of the clinical benefit of standard dose roxadustat.

### Statistical Analysis

The primary efficacy endpoint was compared between the groups using a mixed model for repeated measures considering treatment group, study visit, treatment by visit interaction as fixed effects, and the corresponding endpoint’s baseline value and baseline estimated glomerular filtration rate as covariates. A sensitivity analysis on the primary endpoint was performed using a multiple imputation analysis of covariance model. The primary efficacy analysis was performed in the per-protocol set and full analysis set, with the per-protocol set being the primary analysis population to ensure a conservative approach. For the secondary efficacy endpoints of the proportion of patients with an average hemoglobin (weeks 12–16) of 100 to 120 g/l and the proportion requiring rescue therapy, logistic regression was used to compare the groups. For the secondary efficacy endpoint of hemoglobin variability, the measures were compared by analysis of covariance. All safety assessment data were summarized by collection timepoint. The analysis populations are outlined in the Supplementary Methods. The data were analyzed using SAS software, version 9.4. *P* < 0.05 was considered statistically significant.

## Results

### Demographic and Baseline Characteristics

**Figure 1:**
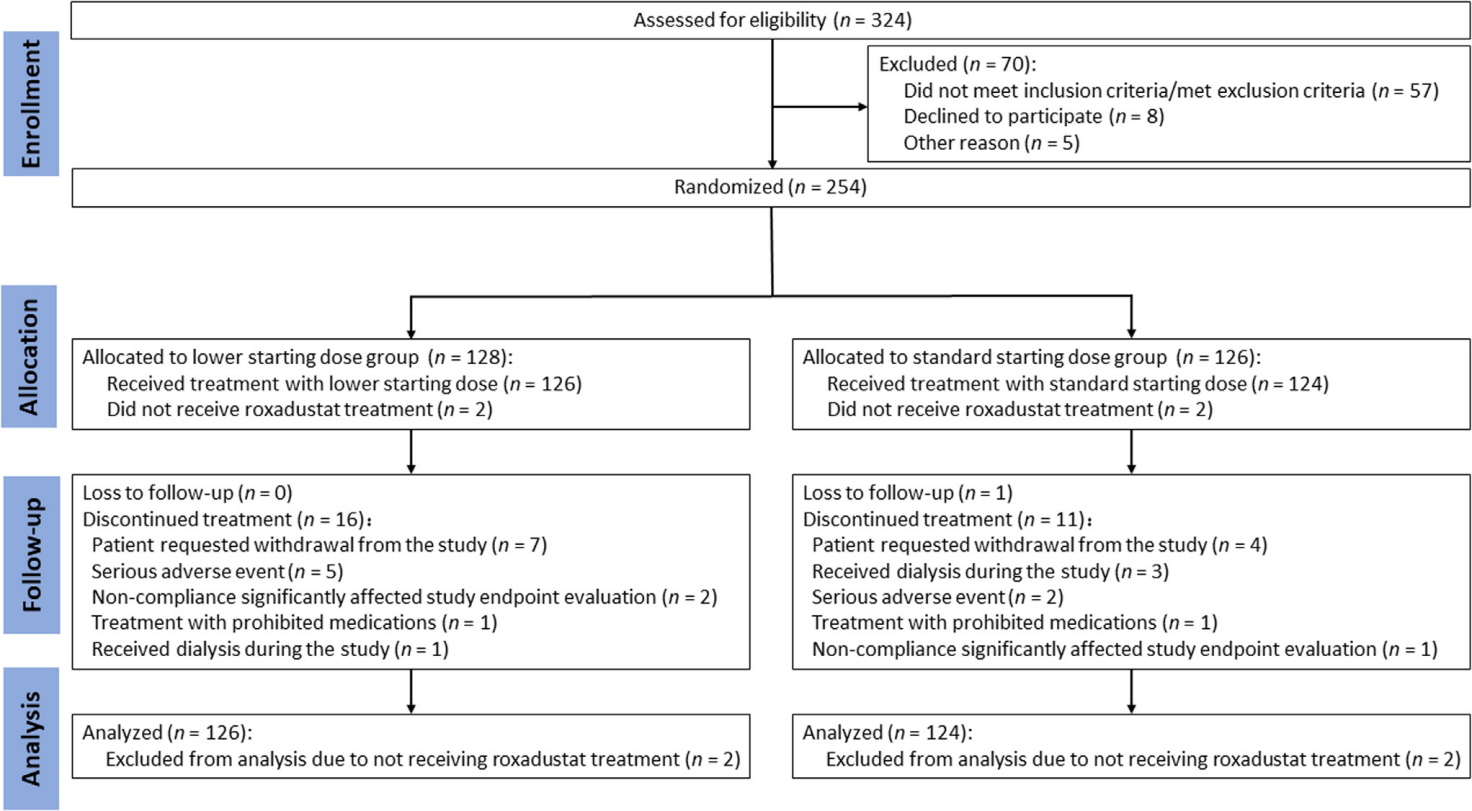
Patient disposition flowchart.

Overall, 324 patients were screened for eligibility, and 254 were successfully randomized (78.4%) (lower: *n* = 128; standard: *n* = 126). In the lower starting dose group, 126 of 128 patients (98.4%) received roxadustat and 112 of 128 (87.5%) completed the study. In the standard starting dose group, 124 of 126 (98.4%) received roxadustat and 114 of 126 (90.5%) completed the study (Figure 1).

The demographic and baseline characteristics were similar between the 2 groups (Table 1). In the lower dose group, 54 patients (42.9%) were male, compared with 63 (51.2%) in the standard dose group. The mean (SD) age was 55.4 (12.58) years in the lower dose group and 55.8 (12.54) years in the standard dose group. The mean (SD) body mass index was 23.43 (3.22) kg/m^2^ and 23.85 (3.56) kg/m^2^ in the lower and standard dose groups, respectively. The leading CKD etiology was primary glomerular disease (86/249 [34.7%]). The median CKD duration was 14.65 (2.14–81.53) months in the lower dose group and 13.01 (2.53–50.23) months in the standard dose group. The mean baseline estimated glomerular filtration rate was 21.2 (12.39) ml/min per 1.73 m^2^ and 20.7 (12.51) ml/min per 1.73 m^2^ in the lower and standard dose groups, respectively. Most patients had stage 4 (39.0%) or 5 (40.2%) CKD. The mean (SD) baseline hemoglobin was 89.42 (6.96) and 90.35 (6.83) g/l, and 8.7% and 7.3% of the patients had a hemoglobin concentration < 80 g/l in the lower and standard dose groups, respectively. The median (Q1, Q3) ferritin concentration was 294.9 (179.8, 434.1) μg/l in the lower dose group and 303.0 (194.4, 525.6) μg/l in the standard dose group, and the median transferrin saturation was 26.85% (21.8%, 34.9%) and 28.82% (22.5%, 36.7%), respectively. In the full analysis set, 13.5% of the patients in the lower dose group had a C-reactive protein or high-sensitivity C-reactive protein concentration greater than the upper limit of normal compared with 13.0% in the standard dose group. The data on previous and concomitant oral and i.v. iron use are provided in Supplementary Table S3.

**Table 1:** Demographic and baseline characteristics of the patients

Characteristics	Lower dose	Standard dose	Overall
Full analysis set	*n* = 126	*n* = 123	*N* = 249
Age, yrs, mean (SD)	55.4 (12.58)	55.8 (12.54)	55.6 (12.54)
Sex, male, *n* (%)	54 (42.9)	63 (51.2)	117 (47.0)
Body weight, kg, mean (SD)	63.67 (11.35)	64.59 (11.19)	64.12 (11.26)
Body mass index, kg/m^2^, mean (SD)	23.43 (3.22)	23.85 (3.56)	23.64 (3.38)
Top 3 most common CKD etiologies, *n* (%)			
Primary glomerular disease	41 (32.5)	45 (36.6)	86 (34.5)
Diabetic kidney disease	46 (36.5)	34 (27.6)	80 (32.1)
Hypertensive renal disease	13 (10.3)	10 (8.1)	23 (9.2)
CKD duration, months, median (Q1, Q3)	14.65 (2.14, 81.53)	13.01 (2.53, 50.23)	13.60 (2.17, 61.86)
eGFR, ml/min per 1.73 m^2^, mean (SD)	21.2 (12.39)	20.7 (12.51)	21.0 (12.43)
Median (Q1, Q3)	18.5 (12, 27)	17 (11, 27)	18.0 (12, 27)
CKD stage, *n* (%)			
3	26 (20.6)	26 (21.1)	52 (20.9)
4	51 (40.5)	46 (37.4)	97 (39.0)
5	49 (38.9)	51 (41.5)	100 (40.2)
Hemoglobin, g/l			
Mean (SD)	89.42 (6.96)	90.35 (6.83)	89.88 (6.90)
≥ 80, *n* (%)	115 (91.3)	114 (92.7)	229 (92.0)
< 80, *n* (%)	11 (8.7)	9 (7.3)	20 (8.0)
Ferritin, μg/l, median (Q1, Q3)	294.90 (179.80, 434.10)	303.00 (194.40, 526.60)	299.90 (184.50, 490.50)
Transferrin saturation, %			
Median (Q1, Q3)	26.85 (21.80, 34.94)	28.82 (22.45, 36.66)	28.07 (22.02, 35.97)
< 5, *n* (%)	1 (0.8)	1 (0.8)	2 (0.8)
5–20, *n* (%)	25 (19.8)	19 (15.4)	44 (17.7)
>20, *n* (%)	98 (77.8)	96 (78.0)	194 (77.9)
Serum iron, μmol/l			
Mean (SD)	13.23 (7.93)	13.03 (8.51)	13.13 (8.20)
Total iron-binding capacity, μmol/l			
Mean (SD)	44.37 (9.16)	42.23 (10.75)	43.33 (9.99)
Transferrin, g/l			
Mean (SD)	2.09 (0.40)	2.00 (0.36)	2.05 (0.38)
CRP/hs-CRP, *n* (%)			
≤ Upper limit of normal	107 (84.9)	105 (85.4)	212 (85.1)
> Upper limit of normal	17 (13.5)	16 (13.0)	33 (13.3)
Safety analysis set	*n* = 126	*n* = 124	*N* = 250
Platelet count, 10^9^/l			
Mean (SD)	208.0 (63.27)	212.2 (77.41)	210.1 (70.52)
While blood cell count, 10^9^/l			
Mean (SD)	6.23 (2.21)	6.61 (2.01)	6.41 (2.12)
iPTH/PTH, *n* (%)			
Normal	34 (27.0)	42 (34.1)	76 (30.5)
< Lower limit of normal	3 (2.4)	2 (1.6)	5 (2.0)
> Upper limit of normal	89 (70.6)	78 (63.4)	167 (67.1)
Urinary albumin-to-creatinine ratio, *n* (%)			
≤ Upper limit of normal	9 (7.1)	5 (4.1)	14 (5.6)
> Upper limit of normal	106 (84.1)	109 (88.6)	215 (86.3)
Medical history, *n* (%)			
Hypertension	116 (92.1)	113 (91.1)	229 (91.6)
Diabetes mellitus	60 (47.6)	53 (42.7)	113 (45.2)
Diabetic retinopathy	26 (20.6)	24 (19.4)	50 (20.0)
Cardio-cerebrovascular disease	55 (43.7)	44 (35.5)	99 (39.6)
Malignancy	4 (3.2)	5 (4.0)	9 (3.6)

CKD, chronic kidney disease; CRP, C-reactive protein; eGFR, estimated glomerular filtration rate; hs-CRP, high-sensitivity C-reactive protein; iPTH, intact parathyroid hormone; PTH, parathyroid hormone; ULN, upper limit of normal.

Patients were tested for either CRP or hs-CRP. When counting the frequency of patients in each group (≤ ULN or > ULN), CRP took priority over hs-CRP. Patients were tested for either iPTH or PTH. When counting the frequency of patients in each group (normal, < LLN, or > ULN), iPTH took priority over PTH. The full analysis set included all randomized patients who received at least one dose of roxadustat and who had at least 1 nonmissing postbaseline hemoglobin value. The safety analysis set consisted of all patients who received at least one dose of roxadustat.

### Efficacy Endpoints

#### Primary Endpoint

**Figure 2:**
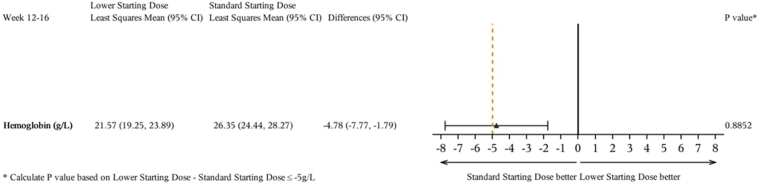
Noninferiority forest plot for the primary efficacy end point (per-protocol set). CI, confidence interval.

In the per-protocol set, the least-squares mean (95% CI) change in hemoglobin from baseline averaged over weeks 12 to 16 was 21.57 (19.25–23.89)g/l in the lower and 26.35 (24.44–28.27)g/l in the standard (difference −4.78 [95% CI:−7.77 to−1.79] g/l) dose groups (Figure 2, Table 2). The 95% CI lower limit was less than the predefined noninferiority margin of −5 g/l; therefore, noninferiority was not concluded. The results were similar in the full analysis set. The results of the sensitivity analysis were consistent with the primary analysis (Supplementary Table S4).

**Table 2:** Mean change in hemoglobin from baseline averaged over weeks 12–16 (per-protocol set and full analysis set)

Visit	Statistic	Lower dose	Standard dose	Treatment difference [95% CI]
Per-protocol set				
Baseline^a^	n	115	111	
	Mean (SD)	89.44 (7.00)	90.59 (6.69)	
Average over weeks 12–16^b^	n	106	108	
	Mean (SD)	111.26 (13.38)	117.12 (9.97)	
Change from baseline in average over weeks 12–16 (MMRM)	LSM [95% CI]	21.57 [19.25–23.89]	26.35 [24.44–28.27]	−4.78 [−7.77 to−1.79]
Full analysis set				
Baseline^a^	n	126	123	
	Mean (SD)	89.42 (6.96)	90.35 (6.83)	
Average over weeks 12–16^b^	n	115	119	
	Mean (SD)	110.42 (13.79)	116.21 (11.06)	
Change from baseline in average over weeks 12–16 (MMRM)	LSM [95% CI]	20.69 [18.35–23.04]	25.57 [23.55–27.60]	−4.88 [−7.96 to −1.79]

CI, confidence interval; LSM, least-squares mean; MMRM, mixed model for repeated measures.

The primary efficacy endpoint was compared using the MMRM with treatment group, visit, and treatment by visit interaction as fixed effects, and baseline hemoglobin and baseline estimated glomerular filtration rate as covariates.

Noninferiority was established if the lower bound of the 2-sided 95% CI for the treatment difference in change from baseline hemoglobin (lower starting dose – standard starting dose) was greater than −5 g/l.

For the noninferiority test, the per-protocol set was the primary analysis population.

a Baseline hemoglobin was defined as the average of the last 2 available values before the first dose of roxadustat.

b Average over weeks 12–16 = (hemoglobin at week 12 + hemoglobin at week 16) ÷ 2.

**Figure 3:**
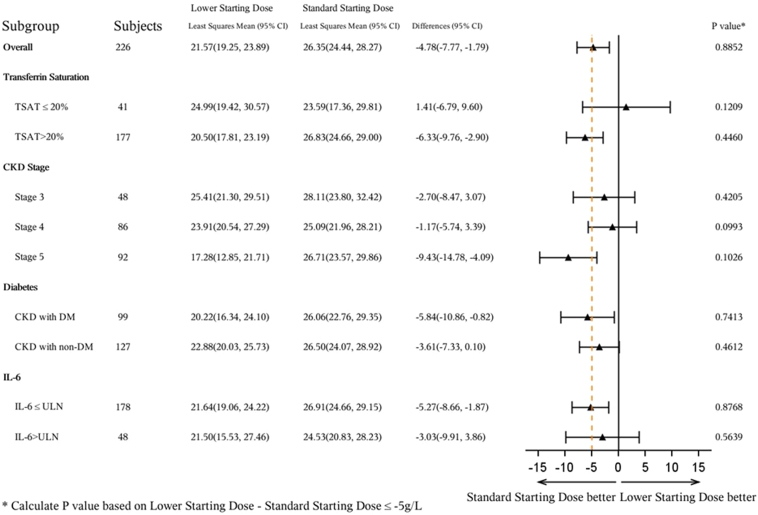
Subgroup analysis forest plot of the mean change in hemoglobin from baseline averaged over weeks 12 to 16 (per-protocol set).

In the subgroup analysis by CKD stage in the lower dose group, the mean hemoglobin change from baseline over weeks 12 to 16 was numerically lower in patients with anemia and stage 5 CKD *(n* = 39) than in those with stage 3 CKD (*n* = 22) or stage 4 CKD (*n* = 45) (stage 3: 25.41 g/l; stage 4: 23.91 g/l; stage 5: 17.28 g/l). The same pattern was not observed in the standard dose group (stage 3 [*n* = 22]: 28.11 g/l; stage 4 [*n* = 41]: 25.09 g/l; stage 5 [*n* = 45]: 26.71 g/l) (Figure 3).

#### Secondary Endpoints

In the full analysis set, there was no difference in the proportion of patients with an average hemoglobin of 100 to 120 g/l over weeks 12 to 16 (Table 3) (lower: 46.0% [58/126] vs. standard dose: 47.2% [58/123]; odds ratio [OR]:1.084 [95% CI: 0.646–1.817]; *P* = 0.7608). The results were similar in the per-protocol set. A *post hoc* analysis was conducted to compare the proportions of patients who achieved hemoglobin of 110 to 130 g/l and 100 to 130 g/l over the same period (Table 3). More patients in the standard dose group achieved an average hemoglobin within these ranges (110–130 g/l, lower: 50.8 % [64/126] vs. standard: 68.3 % [84/123], *P* = 0.0293; 100–130 g/l, lower: 69.8% [88/126] vs. standard: 86.2% [106/123], *P* = 0.0217).

**Table 3:** Proportions of patients who achieved hemoglobin of 100–120, 110–130, and 100–130 g/l over weeks 12–16 (per-protocol set and full analysis set)

Efficacy endpoints	Lower dose	Standard dose	OR (95% CI)^a^	*P* value
Per-protocol set	*n* = 115	*n* = 111		
Average hemoglobin over weeks 12–16, g/l	111.36	117.12		
Patients with hemoglobin of 100–120 g/l over weeks 12–16^b^, *n* (%)	55 (47.8)	53 (47.7)	1.158 (0.671–1.996)	0.5983
Patients with hemoglobin of 110–130 g/l over weeks 12–16^b^, *n* (%)	61 (53.0)	81 (73.0)	0.459 (0.250–0.842)	0.0119
Patients with hemoglobin of 100–130 g/l over weeks 12–16^b^, *n* (%)	84 (73.0)	99 (89.2)	0.386 (0.165–0.904)	0.0283
Full analysis set	*n* = 126	*n* = 123		
Average hemoglobin over weeks 12–16 (g/l)	110.52	116.21		
Patients with hemoglobin of 100–120 g/l over weeks 12–16^b^, *n* (%)	58 (46.0)	58 (47.2)	1.084 (0.646–1.817)	0.7608
Patients with hemoglobin of 110–130 g/l over weeks 12–16^b^, *n* (%)	64 (50.8)	84 (68.3)	0.539 (0.309–0.939)	0.0293
Patients with hemoglobin of 100–130 g/l over weeks 12–16^b^, *n* (%)	88 (69.8)	106 (86.2)	0.424 (0.203–0.882)	0.0217

CI, confidence interval; OR, odds ratio.

The proportion of patients with a hemoglobin of 110–130 g/l and 100–130 g/l over weeks 12–16 was evaluated as a *post hoc* analysis.

a Odds ratio: lower dose group ÷ standard dose group.

b In the logistic regression model, baseline hemoglobin and baseline estimated glomerular filtration rate were included as covariates.

**Figure 4:**
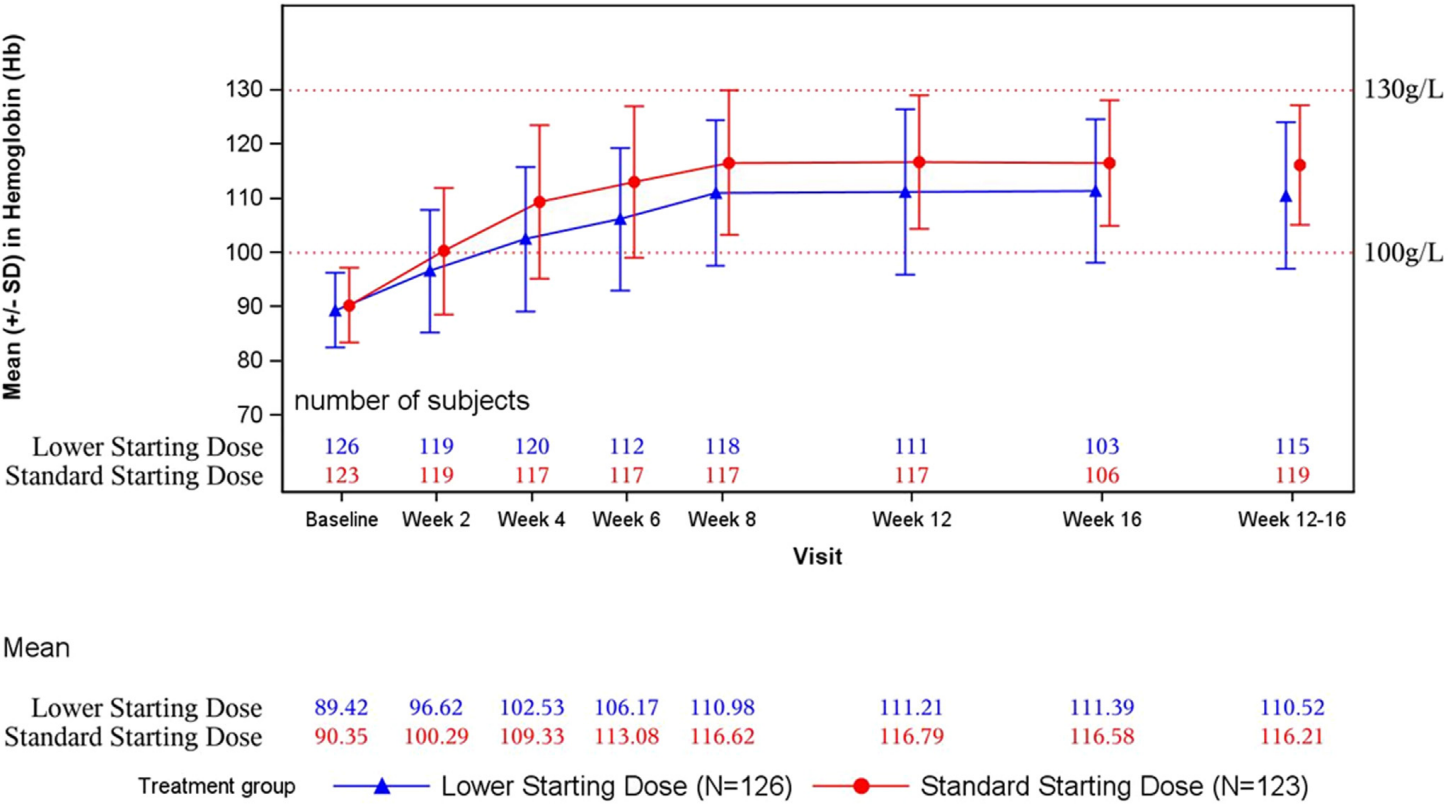
Mean (SD) hemoglobin by visit (full analysis set).

The mean hemoglobin concentrations in both groups by visit are shown in Figure 4. There were no significant differences between the lower and standard dose groups in the mean (SD) proportion of time that hemoglobin was outside the 100 to 130 g/l range from baseline to week 4 (49.2% [44.3%] vs. 43.0% [40.5%], respectively; difference: 3.4% [95% CI:−5.9% to 12.7%]; *P* = 0.4735); baseline to week 8 (40.1% [36.3%] vs. 32.6% [30.5%], respectively; difference 5.3% [95% CI:−2.1% to 12.8%]; *P* = 0.1620); or baseline to week 16 (36.0% [33.7%] vs. 27.6% [26.0%], respectively; difference: 6.9% [95% CI: 0.0%–13.8%]; *P* = 0.0513) (Table 4).

**Table 4:** Proportion of time that hemoglobin was < 100 g/l or > 130 g/l, absolute rate of change in hemoglobin, and change in hemoglobin linear regression slope (full analysis set)

Hemoglobin variability	Lower dose *n* = 126	Standard dose *n* = 123	Treatment difference [95% CI]	
Time with hemoglobin outside 100–130 g/l (%)				*P* value^a^
Baseline to week 4, *n*	123	121		
Mean (SD)	49.2 (44.3)	43.0 (40.5)	3.4 [−5.9 to 12.7]	0.4735
Baseline to week 8, *n*	124	121		
Mean (SD)	40.1 (36.3)	32.6 (30.5)	5.3 [−2.1 to 12.8]	0.1620
Baseline to week 16, *n*	126	123		
Mean (SD)	36.0 (33.7)	27.6 (26.0)	6.9 [−0.0 to 13.8]	0.0513
Summed absolute rate of change in hemoglobin^b^				*P* value^c^
Baseline to week 4, *n*	126	123		
Mean (SD)	2.23 (1.40)	2.61 (1.51)	−0.40 [−0.76 to −0.04]	0.0306
Baseline to week 8, n	126	123		
Mean (SD)	3.00 (1.68)	3.47 (1.76)	−0.49 [−0.92 to−0.06]	0.0264
Baseline to week 16, *n*	126	123		
Mean (SD)	2.92 (1.43)	3.376 (1.47)	−0.49 [−0.85 to−0.13]	0.0079
Hemoglobin linear regression slope^d^				*P* value^e^
Baseline to week 4, *n*	123	121	-	-
Mean (SD)	3.30 (3.00)	4.69 (3.17)	-	-
Week 4 to week 8, *n*	118	120	-	-
Mean (SD)	2.09 (2.74)	1.64 (3.38)	-	-
Week 8 to week 16, *n*	113	116	-	-
Mean (SD)	−0.16 (1.86)	−0.14 (1.63)	-	-
Sum of changes in the slope of linear regression of hemoglobin values^e^				
*n*	112	116		
Mean (SD)	7.13 (4.16)	7.66 (4.66)	−0.62 [−1.78 to 0.55]	0.2972

CI, confidence interval.

a Proportion of time was compared between the groups by analysis of covariance with baseline hemoglobin and baseline estimated glomerular filtration rate as covariates.

b Nonlinear regression was fitted on all hemoglobin data points over each period specified above, with third-degree polynomials, and then the rates of change (slopes) of the trajectory were calculated from the trajectory by taking derivatives.

c The absolute rate of change in hemoglobin was compared between the groups using analysis of covariance with treatment, baseline hemoglobin, and estimated glomerular filtration rate as covariates.

d The linear regression slope of hemoglobin over different time periods in each treatment group was estimated by ordinary least squares.

e The sum of changes in the linear regression slope of hemoglobin was compared between the groups using analysis of covariance with treatment, baseline hemoglobin, and baseline estimated glomerular filtration rate as covariates. The change was defined as the absolute difference between the 2 slopes over adjacent time periods.

The mean (SD) summed absolute rate of change in hemoglobin was slower in the lower dose than in the standard dose group from baseline to week 4 (2.225 [1.404] vs. 2.611 [1.507], respectively; difference:−0.40 [95% CI:−0.76 to−0.04]; *P* = 0.0306); baseline to week 8 (2.998 [1.678]vs. 3.467 [1.761], respectively; difference:−0.49 [95% CI: −0.92 to −0.06]; *P* = 0.0264); and baseline to week 16 (2.917 [1.426]vs. 3.376 [1.468], respectively; difference:−0.49 [95% CI:−0.85 to−0.13]; *P* = 0.0079) (Table 4). The trajectories of change in hemoglobin for the first 9 patients in each group are shown in Supplementary Figure S1.

The mean (SD) hemoglobin linear regression slopes in the lower and standard dose groups, respectively, were 3.302 (2.995) and 4.689 (3.166) from baseline to week 4, 2.087 (2.736) and 1.638 (3.377) from weeks 4 to 8, and −0.160 (1.863) and −0.140 (1.629) from weeks 8 to 16 (Table 4). The mean (SD) summed change in the hemoglobin linear regression slope was numerically lower in the lower dose group (7.131 [4.162]vs. 7.662 [4.664]); however, the intergroup difference was not significant (−0.62 [95% CI:−1.78–0.55]; *P* = 0.2972).

Three patients (2.4%) in the lower dose group and 2 (1.6%) in the standard dose group received rescue therapy during treatment (*P* = 0.4862) (Table 5). Of the 3 patients in the lower dose group, 1 received an ESA, 1 received i.v. iron, and 1 received an ESA and red blood cell transfusion. In the standard dose group, 1 received an ESA and 1 received i.v. iron.

**Table 5:** Rescue therapy (red blood cell transfusion, i.v. iron, or erythropoiesis-stimulating agent) (full analysis set)

Rescue therapy	Lower dose *n* = 126	Standard dose *n* = 123	OR [95% CI]^a^	*P* value
Patients with rescue therapy during the treatment period, *n* (%)	3 (2.4)	2 (1.6)	1.96 [0.30–12.93]	0.4862


			HR [95% CI]^b^	*P* value
Time to first rescue therapy from date of the first dose (days)				
Number of patients censored, *n* (%)	123 (97.6)	121 (98.4)		
Median [95% CI]	NE	NE	1.84 [0.34–9.84]	0.4770

CI, confidence interval; HR, hazard ratio; NE, not estimated; OR, odds ratio.

If a patient did not receive rescue therapy before the end of treatment, the censoring date was the end of treatment.

a The proportion of patients was determined based on the logistic regression model adjusting for baseline estimated glomerular filtration rate and baseline hemoglobin, with the OR and 2-sided 95% CI.

b The comparison of the time to rescue therapy was performed using the Cox proportional-hazards model adjusted for continuous baseline hemoglobin and baseline estimated glomerular filtration rate.

### Exploratory Endpoints

**Figure 5:**
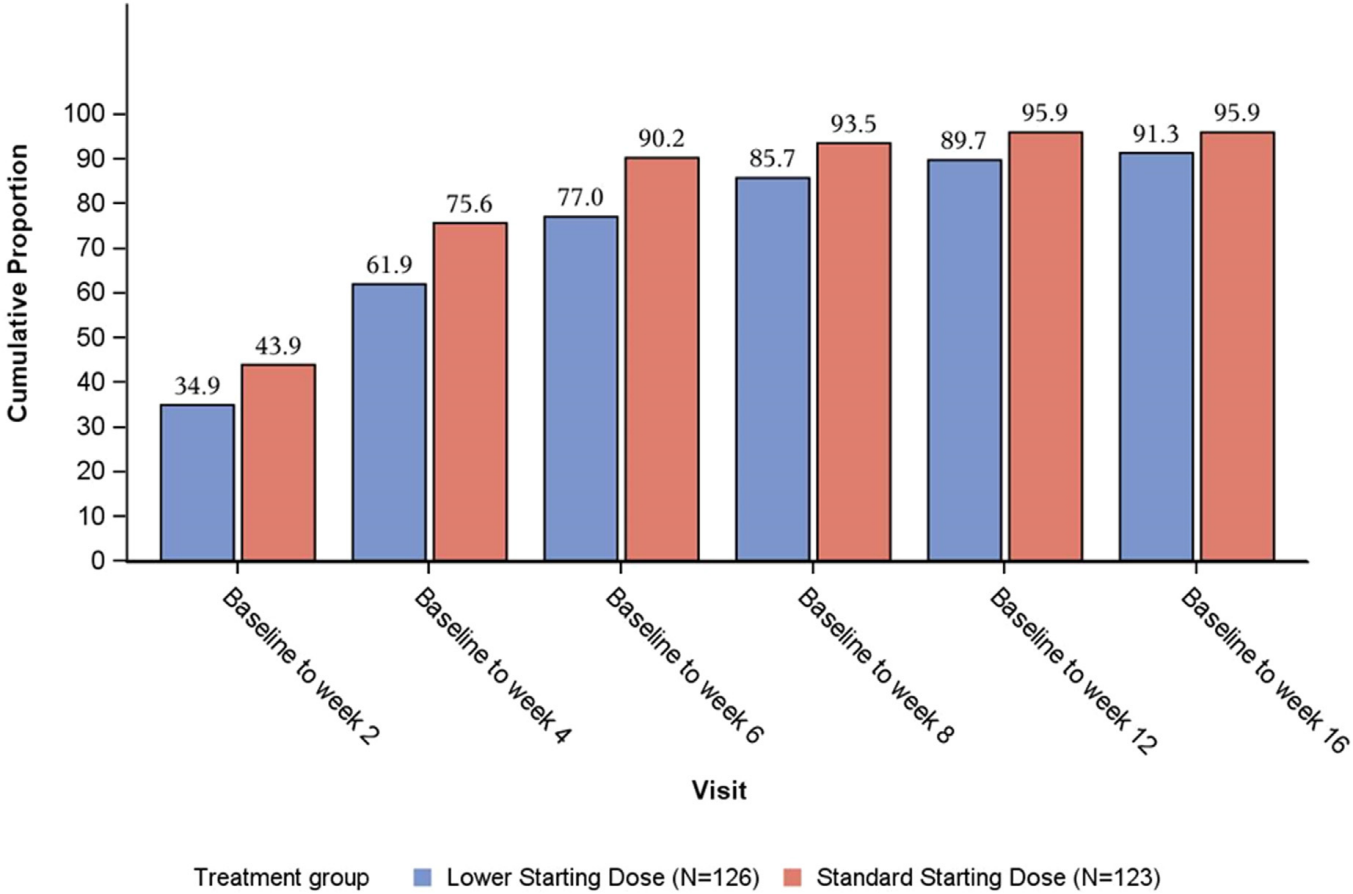
Cumulative proportion of patients with a hemoglobin response (increase of ≥10 g/l) (full analysis set).

**Figure 6:**
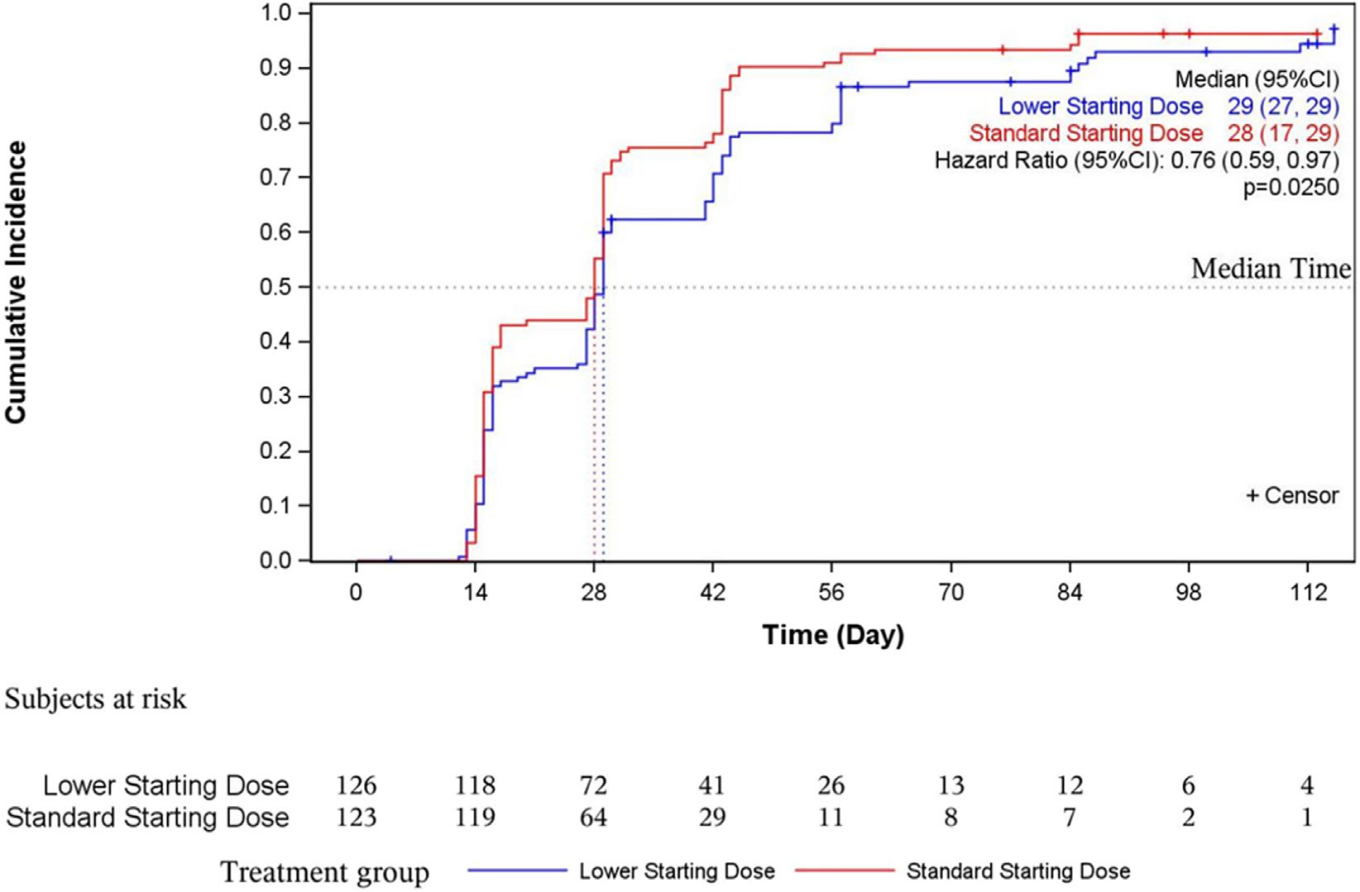
Cumulative probability of first achieving a hemoglobin response (increase of ≥ 10 g/l) from Kaplan–Meier estimates (full analysis set). CI, confidence interval.

In the full analysis set, the cumulative proportion with a hemoglobin response at week 16 (increase of ≥ 10 g/l) was not significantly different (lower: 91.3%; standard: 95.9%; OR: 0.436 [95% CI: 0.146–1.302]; *P* = 0.1370), with > 90% of patients achieving a hemoglobin response and anemia correction in both groups (Figure 5, Supplementary Table S5). In Figure 6, we show the cumulative probability of first achieving a hemoglobin increase ≥ 10 g/l from Kaplan-Meier estimates. The lower dose group took a median of 1 day longer to achieve a hemoglobin increase of ≥ 10 g/l (lower: 29.0 [95% CI: 27.0–29.0] days vs. standard: 28.0 [95% CI: 17.0–29.0] days; hazard ratio: 0.756 [95% CI: 0.592–0.965]; *P* = 0.025).

There was no significant difference in the mean number of dose adjustments per patient (lower: 2.0 [0.90] vs. standard: 1.9 [0.98]; OR: 1.03 [95% CI: 0.86–1.23]; *P* = 0.7290) during treatment; however, the total number of dose increases was significantly higher in the lower dose group (100 vs. 55; *P* = 0.0011) (Supplementary Table S6). The lower dose group had fewer dose suspensions because of hemoglobin overcorrection (0.2 [0.40] vs. 0.3 [0.51]; OR: 0.53 [95% CI: 0.32–0.90]; *P* = 0.0175) and more dose increases (0.8 [0.92]vs. 0.4 [0.73]; OR: 1.74 [95% CI: 1.25–2.41]; *P* = 0.0011) per patient.

### Drug Exposure

The mean (SD) exposure duration was 14.4 (3.5) weeks in the lower dose group and 14.1 (3.7) weeks in the standard dose group. The mean (SD) weekly dose through the overall treatment period was lower in the lower dose group (186.14 [64.71] mg vs. 228.70 [75.82] mg). The median total cumulative dose was 2547.86 mg in the lower dose group and 2929.29 mg in the standard dose group (Supplementary Table S7). The weekly dose of roxadustat by visit is shown in Supplementary Table S8.

### Safety Results

Overall, 170 patients (68.0%) reported TEAEs (lower: 91 [72.2%] vs. standard: 79 [63.7%]) (Table 6). The TEAEs with an incidence ≥ 5% in the lower dose group by preferred term were hyperkalemia, COVID-19, upper respiratory tract infection, hypertension, CKD (aggravation or progression or worsening), and iron deficiency. In the standard dose group, the TEAEs with an incidence ≥ 5% by preferred term were hyperkalemia, COVID-19, hyperphosphatemia, and iron deficiency. TEAEs leading to death occurred in 3 patients (1.2%) (lower: 2 [1.6%] vs. standard: 1 [0.8%]), all cases of which were considered unrelated to roxadustat by the investigator. The number (%) of patients with roxadustat-related TEAEs and treatment-emergent serious adverse events are shown in Table 6, and those with adverse events of special interest are shown in Supplementary Table S9. No patients experienced venous thromboembolism, secondary hypothyroidism, or seizures.

**Table 6:** Overview of TEAEs (safety analysis set)

Safety endpoint	Lower dose *n* = 126 *n* (%)	Standard dose *n* = 124 *n* (%)	Overall *N* = 250 *n* (%)
Any TEAEs	91 (72.2)	79 (63.7)	170 (68.0)
Drug-related TEAEs	5 (4.0)	3 (2.4)	8 (3.2)
TEAEs with severity ≥ Grade 3^a^	38 (30.2)	22 (17.7)	60 (24.0)
TESAEs	31 (24.6)	13 (10.5)	44 (17.6)
TEAEs leading to treatment or study discontinuation	5 (4.0)	2 (1.6)	7 (2.8)
TEAE leading to death	2 (1.6)	1 (0.8)	3 (1.2)
All reported deaths	2 (1.6)	1 (0.8)	3 (1.2)
TEAEs reported by ≥5% of patients in either treatment group^b^			
System Organ Class			
Preferred Term			
Metabolism and nutrition disorders	30 (23.8)	28 (22.6)	58 (23.2)
Hyperkalemia	20 (15.9)	17 (13.7)	37 (14.8)
Hyperphosphatemia	6 (4.8)	8 (6.5)	14 (5.6)
Iron deficiency	7 (5.6)	7 (5.6)	14 (5.6)
Infections and infestations	24 (19.0)	20 (16.1)	44 (17.6)
COVID-19	16 (12.7)	14 (11.3)	30 (12.0)
Upper respiratory tract infection	9 (7.1)	6 (4.8)	15 (6.0)
Vascular disorders	9 (7.1)	6 (4.8)	15 (6.0)
Hypertension	9 (7.1)	6 (4.8)	15 (6.0)
Renal and urinary disorders	9 (7.1)	3 (2.4)	12 (4.8)
Chronic kidney disease	9 (7.1)	3 (2.4)	12 (4.8)

Drug-related, possibly related or related; MedDRA, medical dictionary for regulatory activities; TEAEs, treatment-emergent adverse events; TESAEs, treatment-emergent serious adverse events.

a Grade ≥ 3 was defined according to the Common Terminology Criteria for Adverse Events.

b TEAEs reported by ≥ 5% of patients in either treatment group were coded by MedDRA Version 25.1 System Organ Class and Preferred Term.

## Discussion

In this dose-optimization study, although noninferiority of the lower dose was not established for the primary efficacy endpoint of mean change in hemoglobin from baseline over weeks 12 to 16, mean hemoglobin increased by 10 to 20 g/l from baseline to week 4 in both groups. Moreover, the least-squares mean change in hemoglobin from baseline over weeks 12 to 16 was > 20 g/l in both groups, illustrating that both doses were effective at correcting anemia in the NDD CKD population.

A hemoglobin response was achieved in > 90% of patients in both groups at week 16, similar to previous studies from other countries. In a phase 2b study,^14^ 91.3% of patients treated with roxadustat at a fixed starting dose of 50 mg TIW achieved a hemoglobin response at week 24. In another study, the response rates were 81.5%, 100%, and 100% with 50, 70, and 100 mg roxadustat TIW, respectively.^15^ In a phase 3 study, the hemoglobin response rate was 97.0% overall (50 mg: 93.9%; 70 mg: 100%).^16^ These findings support the clinical value of both lower and standard starting dose roxadustat for anemia correction in CKD.

In the subgroup analysis by CKD stage, the mean change in hemoglobin from baseline over weeks 12 to 16 in those treated with the lower starting dose was less for patients with anemia in stage 5 CKD than for those with anemia in stage 3 or 4 CKD, potentially contributing to the lack of demonstration of noninferiority of the lower starting dose. This suggests that the lower starting dose may be less effective at increasing hemoglobin in patients with stage 5 CKD, indicating that the current approved standard starting dose^18^ should be retained for these patients, whereas the 1-step-lower starting dose may be useful for patients with stage 3 or 4 CKD, thereby providing important evidence for clinical practice.

Moreover, although there was no significant difference between the lower and standard starting dose groups in the proportion of patients with mean hemoglobin of 100 to 120 g/l at weeks 12 to 16, which is the recommended target in the roxadustat approved product insert,^18^ the *post hoc* analysis showed that the proportion with hemoglobin of 110 to 130 g/l (recommended therapeutic target in Chinese Guideline^21^) or 100 to 130 g/l averaged over weeks 12 to 16 was significantly lower in the lower dose group. This suggests that the standard dose may be more effective for achieving certain hemoglobin targets.

In terms of hemoglobin variability, we observed no significant difference in the change in hemoglobin linear regression slope or in the proportion of time that hemoglobin was outside of the threshold range of 100 to 130 g/l between the lower and standard doses. However, a significant difference was observed in the summed absolute rate of change in hemoglobin, suggesting that the magnitude of hemoglobin variability was smaller in the lower dose group. This is important because previous studies have demonstrated a higher incidence of adverse clinical events and mortality in patients with CKD with greater hemoglobin variability.^22^, ^23^, ^24^ Interestingly, although hemoglobin fluctuated less in the lower starting dose group than in the standard starting dose group, the overall and drug-related TEAEs were no different between the groups. However, given its association with adverse clinical events, long-term hemoglobin variability should be compared between lower and standard roxadustat starting doses in future global studies.

Roxadustat showed an acceptable safety profile, with adverse events being typical of the population with NDD stage 3 to 5 CKD and consistent with the known safety profile of roxadustat.^25^^,^^26^ Patient enrollment was underway during the COVID-19 pandemic, which explains why COVID-19 was among the top TEAEs. The proportion of patients who experienced TEAEs was numerically higher in the lower dose group than in the standard dose group (72.2% vs. 63.7%); however, this may have occurred by chance, and no new safety concerns were identified. Nevertheless, we did not observe a better safety profile with lower starting dose roxadustat.

Finally, the fact that the lower starting dose group achieved the target hemoglobin with less hemoglobin fluctuation and less roxadustat exposure may suggest a possible cost saving for the Chinese government and for patients. We suggest that the lower starting dose is appropriate for patients with anemia in NDD stage 3 or 4 CKD, but not stage 5. However, it is important to consider that the lower starting dose group reached the hemoglobin target with twice as many dose-increases as the standard starting dose group (100 vs. 55; *P* = 0.0011), and the safety profile of the lower starting dose was not better than that of the standard starting dose. These findings could help physicians to decide on the appropriate roxadustat starting dose for individual patients with anemia in CKD in clinical practice.

### Limitations

We only used temporal methods to evaluate hemoglobin variability. Nontemporal measures, such as the SD of hemoglobin values and the coefficient of hemoglobin variation, should be considered in future studies.^17^ The influence of hemoglobin variability over 16 weeks on long-term health outcomes was not evaluated. The study only involved Chinese individuals, so the findings may not be generalizable to other populations. Nevertheless, both starting doses of roxadustat were effective for anemia correction, and the lower starting dose demonstrated less hemoglobin fluctuation. These results support the use of different starting doses of roxadustat in clinical practice and suggest that an update to the approved product package insert may be warranted in China.

## Conclusion

In patients with anemia in NDD stage 3 to 5 CKD, lower starting dose roxadustat did not demonstrate noninferiority to standard starting dose roxadustat in terms of the hemoglobin increase in patients with stage 3 to 5 CKD without dialysis, and a comparable proportion achieved the hemoglobin target (100–120 g/l). The lower starting dose did not demonstrate a better safety profile. Both starting doses were effective for anemia correction, which is of clinical value. The lower starting dose had less hemoglobin fluctuation and is recommended for patients with CKD stage 3 to 4.

## Appendix

### List of the members of the FGCL-4592-858 Working Group

Xiangmei Chen, Guangyan Cai, Ping Li, Xuefeng Sun, Li Zhang, Hongli Lin, Niansong Wang, Yuehong Li, Sumei Zhao, Ping Fu, Hong Cheng, Zhiyong Guo, Wanhong Lu, Yani He, Fengmin Shao, Qiang He, Shiren Sun, Wei Liang, Hongtao Yang, Zhaohui Ni, Qiongqiong Yang, Wenge Li, Aihua Zhang, Guojuan Zhang, Gengru Jiang, Bo Lin, Yanning Zhang, Wenhu Liu, Yonghui Mao, Jinsheng Xu, Weiping Liu, Song Wang, Xiaodong Zhang, Jurong Yang, Hongwei Jiang, Yiqing Wu, Cuihua Huang, and Shuting Pan.

## Disclosure

YW, CH, and SP are employees of FibroGen China’s Medical Affairs and Clinical Biometrics Department. All the other authors declared no competing interests.

## Supplementary Material

Supplementary File (PDF) **Supplementary Methods. Figure S1.** Fitted trajectory of the hemoglobin changes of the first nine patients by treatment group (full analysis set). **Table S1.** Additional inclusion and exclusion criteria. **Table S2.** Guidelines for roxadustat dose adjustment. **Table S3.** Prior and concomitant oral and i.v. iron therapy (safety analysis set). **Table S4.** Sensitivity analysis of the mean change in hemoglobin from baseline averaged over weeks 12 to 16 (per-protocol set and full analysis set). **Table S5.** Proportion of patients achieving a hemoglobin response (full analysis set). **Table S6.** Roxadustat dose adjustment throughout the treatment period (full analysis set). **Table S7.** Study drug exposure (safety analysis set). **Table S8.** Weekly roxadustat dose by visit (full analysis set). **Table S9.** Summary of adverse events of special interest (safety analysis set). **Table S10.** Criteria for assessing major protocol deviations. **Table S11.** Summary of serum iron parameters and changes from baseline by visit (full analysis set).

## Footnotes

Supplementary File (PDF)

**Supplementary Methods.**
**Figure S1.** Fitted trajectory of the hemoglobin changes of the first nine patients by treatment group (full analysis set). **Table S1.** Additional inclusion and exclusion criteria. **Table S2.** Guidelines for roxadustat dose adjustment. **Table S3.** Prior and concomitant oral and i.v. iron therapy (safety analysis set). **Table S4.** Sensitivity analysis of the mean change in hemoglobin from baseline averaged over weeks 12 to 16 (per-protocol set and full analysis set). **Table S5.** Proportion of patients achieving a hemoglobin response (full analysis set). **Table S6.** Roxadustat dose adjustment throughout the treatment period (full analysis set). **Table S7.** Study drug exposure (safety analysis set). **Table S8.** Weekly roxadustat dose by visit (full analysis set). **Table S9.** Summary of adverse events of special interest (safety analysis set). **Table S10.** Criteria for assessing major protocol deviations. **Table S11.** Summary of serum iron parameters and changes from baseline by visit (full analysis set).
**CONSORT checklist.**
