# Supplementary material for: Randomized Trial of Lower-Dose Roxadustat Efficacy and Safety in Non–Dialysis-Dependent CKD-Associated Anemia
Source: Kidney Int Rep. 2025 Jan 27;10(4):1050–62. doi: 10.1016/j.ekir.2025.01.027 (PMC12034882; doi:10.1016/j.ekir.2025.01.027)
Supplement: Supplementary File (PDF) — Supplementary Methods. Figure S1. Fitted trajectory of the hemoglobin changes of the first nine patients by treatment group (full analysis set). Table S1. Additional inclusion and exclusion criteria. Table S2. Guidelines for roxadustat dose adjustment. Table S3. Prior and concomitant oral and i.v. iron therapy (safety analysis set). Table S4. Sensitivity analysis of the mean change in hemoglobin from baseline averaged over weeks 12 to 16 (per-protocol set and full analysis set). Table S5. Proportion of patients achieving a hemoglobin response (full analysis set). Table S6. Roxadustat dose adjustment throughout the treatment period (full analysis set). Table S7. Study drug exposure (safety analysis set). Table S8. Weekly roxadustat dose by visit (full analysis set). Table S9. Summary of adverse events of special interest (safety analysis set). Table S10. Criteria for assessing major protocol deviations. Table S11. Summary of serum iron parameters and changes from baseline by visit (full analysis set). [file mmc1.pdf]

Randomized Trial of Lower-Dose Roxadustat Efficacy/Safety in Non-Dialysis-Dependent Chronic Kidney Disease-Associated Anemia

Ping Li et al.

Supplementary Material

Appendix. FGCL-4592-858 Working Group members

Supplementary Methods. Primary Efficacy Analysis, Sensitivity Analysis, Analysis Populations, Roxadustat Dose Adjustment Guidance, Supplemental Iron Use, Rescue Therapy, Adverse Events of Special Interest

Supplementary Figure S1. Fitted trajectory of the hemoglobin changes of the first nine patients by treatment group (full analysis set)

Supplementary Table S1. Additional inclusion and exclusion criteria

Supplementary Table S2. Guidelines for roxadustat dose adjustment

Supplementary Table S3. Prior and concomitant oral and intravenous iron therapy (safety analysis set)

Supplementary Table S4. Sensitivity analysis of the mean change in hemoglobin from baseline averaged over weeks 12–16 (per-protocol set and full analysis set)

Supplementary Table S5. Proportion of patients achieving a hemoglobin response (full analysis set)

Supplementary Table S6. Roxadustat dose adjustment throughout the treatment period (full analysis set)

Supplementary Table S7. Study drug exposure (safety analysis set)

Supplementary Table S8. Weekly roxadustat dose by visit (full analysis set)

Supplementary Table S9. Summary of AESI (safety analysis set)

Supplementary Table S10. Criteria for assessing major protocol deviations

Supplementary Table S11. Summary of serum iron parameters and changes from baseline by visit (full analysis set)

CONSORT checklist.

Appendix. FGCL-4592-858 Working Group Members

FGCL-4592-858 Working Group members: Xiangmei Chen[1], Guangyan Cai[1], Ping Li[1], Xuefeng Sun[1], Li Zhang[1], Hongli Lin[2], Niansong Wang[3], Yuehong Li[4], Sumei Zhao[5], Ping Fu[6], Hong Cheng[7], Zhiyong Guo[8], Wanhong Lu[9], Yani He[10], Fengmin Shao[11], Qiang He[12], Shiren Sun[13], Wei Liang[14], Hongtao Yang[15], Zhaohui Ni[16], Qiongqiong Yang[17], Wenge Li[18], Aihua Zhang[19], Guojuan Zhang[20], Gengru Jiang[21], Bo Lin[22], Yanning Zhang[23], Wenhu Liu[24], Yonghui Mao[25], Jinsheng Xu[26], Weiping Liu[27], Song Wang[28], Xiaodong Zhang[29], Jurong Yang[30], Hongwei Jiang[31], Yiqing Wu[32], Cuihua Huang[32], Shuting Pan[32]

[1]Department of Nephrology, First Medical Center of the Chinese PLA General Hospital, Nephrology Institute of the Chinese People's Liberation Army, National Key Laboratory of Kidney Diseases, National Clinical Research Center for Kidney Diseases, Beijing Key Laboratory of Kidney Disease Research, Beijing China; [2]Department of Nephrology, The First Affiliated Hospital of Dalian Medical University, Dalian, China; [3]Department of Nephrology Shanghai Sixth People's Hospital Affiliated to Shanghai JiaoTong University, Shanghai, China; [4]Department of Nephrology, Beijing Tsinghua Changgung Hospital, Clinical Medicine of Tsinghua University, Beijing, China; [5]Department of Nephrology, Beijing Chao-Yang Hospital, Capital Medical University; [6]Department of Nephrology, West China Hospital, Sichuan University, Chengdu, China; [7]Department of Nephrology, Beijing Anzhen Hospital, Capital Medical University, Beijing, China; [8]Department of Nephrology, The First Affiliated Hospital of Naval Medical University, Changhai Hospital, Shanghai, China; [9]The First Affiliated Hospital of Xi'an JiaoTong University, Xi'an, China; [10]Department of Nephrology, Army Medical Center of Chinese People's Liberation Army, Chongqing, China; [11]Department of Nephrology, Henan Provincial People's Hospital, Zhengzhou, China; [12]Department of

Nephrology, The First Affiliated Hospital of Zhejiang Chinese Medical University, Zhejiang Provincial Hospital of Traditional Chinese Medicine, Hangzhou, China; [13]Department of Nephrology, The First Affiliated Hospital of PLA Air Force Military Medical University, Xi'an, China; [14]Department of Nephrology, Renmin Hospital of Wuhan University, Wuhan, China; [15]First Teaching Hospital of Tianjin University of Traditional Chinese Medicine, Tianjin, China; [16]Department of Nephrology, Renji Hospital Affiliated to Shanghai Jiaotong University School of Medicine, Shanghai, China; [17]Department of Nephrology, Sun Yat-sen Memorial Hospital, Sun Yat-sen University, Guangzhou, China; [18]Department of Nephrology, China-Japan Friendship Hospital, Beijing, China; [19]Department of Nephrology, Xuanwu Hospital, Capital Medical University, Beijing, China; [20]Department of Nephrology, Beijing Tongren Hospital, Capital Medical University, Beijing, China; [21]Department of Nephrology, Xinhua Hospital Affiliated to Shanghai JiaoTong University School of Medicine, Beijing, China; [22]Department of Nephrology, Zhejiang Provincial People's Hospital, Hangzhou, China; [23]Department of Nephrology, General Hospital of Northern Theater Command of PLA, Shenyang, China; [24]Department of Nephrology, Beijing Friendship Hospital, Capital Medical University, Beijing, China; [25]Department of Nephrology, Beijing Hospital, Beijing, China; [26]Department of Nephrology, The Fourth Hospital of Hebei Medical University, Shijiazhuang, China; [27]Department of Nephrology, The First Hospital of Qinhuangdao, Qinhuangdao, China; [28]Department of Nephrology, Peking University Third Hospital, Beijing, China; [29]Department of Nephrology, The First Hospital of Shanxi Medical University, Taiyuan, China; [30]Department of Nephrology, The Third Affiliated Hospital of Chongqing Medical University, Chongqing, China; [31]Department of endocrinology, The First Affiliated Hospital of Henan University of Science and Technology, Luoyang, China; [32]Medical Affairs and Clinical

Biometrics Department, FibroGen (China), Medical Technology Development Company Ltd.,

Beijing, China

Supplementary Methods

Primary Efficacy Analysis

The hypothesis to be tested for the primary efficacy analysis was as follows:

H0: The difference in mean hemoglobin change from baseline to the average over weeks 12–16 between the lower starting dose and the standard starting dose groups will be ≤−0.5 g/dL vs. H1: The difference in mean hemoglobin change from baseline to the average over weeks 12–16 between the lower starting dose and the standard starting dose group will be >−0.5 g/dL.

The primary efficacy analysis tested the non-inferiority of the lower starting dose to the standard starting dose, with non-inferiority concluded if the lower bound of the two-sided 95% confidence interval (CI) for the treatment difference in change from baseline hemoglobin (lower starting dose − standard starting dose) was >−0.5 g/dL. The primary efficacy endpoint was compared using the mixed model for repeated measures with treatment group, visit, and the treatment by visit interaction as fixed effects, and the corresponding endpoint's baseline value and baseline estimated glomerular filtration rate as covariates. An unstructured variance-covariance structure was used to model the within-patient errors. This variance-covariance matrix was estimated across the treatment groups. If the model failed to converge, the covariance structure that converged with the highest logarithmic restricted maximum likelihood was used. The *p*-value and two-sided 95% CIs for the treatment group means and the treatment difference in the least-squares mean over weeks 12–16 were determined based on the results from the mixed model for repeated measures.

The primary efficacy analysis was performed for the per-protocol set (PPS) and full analysis set (FAS), with the PPS being the primary analysis population.

The hemoglobin results obtained from a local laboratory were used for all efficacy analyses.

Sensitivity Analysis

A sensitivity analysis on the primary endpoint was conducted on the FAS and PPS using Multiple Imputation Analysis of Covariance (MI ANCOVA) according to the following steps:

1. We generated 200 datasets using seed 224605 where intermittent missing hemoglobin data were imputed for each treatment group based on the non-missing hemoglobin data, baseline hemoglobin levels, and baseline eGFR from all patients within each starting dose group using the Monte Carlo Markov Chain (MCMC) imputation model. The MCMC statement in the SAS PROC MI procedure with MONOTONE option was used. Option chain=multiple was also used to ensure a separate chain for each imputation. As a result, each dataset had only one missing data value or a monotone missing data pattern.
2. For each dataset from step 1, missing end data (hemoglobin up to the end of the evaluation period) was imputed using seed 419753, resulting in 200 complete imputed datasets. Missing data at week 4 were imputed using the regression imputation model with hemoglobin from week 2 using the SAS PROC MI procedure with the REGRESSION option in the MONOTONE statement. The SAS PROC MI procedure used data separately from the different treatment allocations to impute the missing data for a specific week (i.e., only those that needed imputation for that week). As patients with complete records for that week were excluded from this step, they did not contribute to that week's imputation. This was repeated for all other scheduled weeks sequentially (week 2 to the end of the evaluation period). Patients whose missing data were imputed for previous weeks contributed to the imputation for subsequent weeks.

The regression imputation model included an intercept, the hemoglobin slopes from previous weeks, and the stratification factors. Categorical variables were clarified in the CLASS statement.

3. ANCOVA was performed for each of the 200 datasets where the average of the observed and imputed hemoglobin values between weeks 12–16 for each patient was taken as the dependent variable, baseline hemoglobin and estimated glomerular filtration rate as covariates, and treatment allocation as a fixed effect.

4. Estimates from the results of each of the 200 ANCOVA runs using SAS PROC MIANALYZE were combined. Hemoglobin averaged over weeks 12–16 was calculated based on the observed and imputed hemoglobin values at weeks 12 and 16. The treatment effect estimate (e.g., least-squares mean of the difference in hemoglobin between the different starting dose groups) and its two-sided 95% CI with corresponding *p*-value were calculated.

Analysis Populations

The safety analysis set included all patients treated with ≥1 dose (*n* = 250). The FAS included all randomized patients treated with ≥1 dose with at least one post-baseline hemoglobin measurement (*n* = 249), including 126 in the lower dose group and 123 in the standard dose group. The PPS included all patients in the FAS treated with roxadustat for ≥2 weeks without major protocol deviations that would significantly influence the primary endpoint evaluation, which were pre-specified prior to database lock and final study analyses (**Supplementary Table S10**). Twenty-three patients in the FAS were excluded from the PPS because of major protocol deviations (*n* = 18) or a treatment duration of <2 weeks (*n* = 5). Therefore, the PPS comprised 226 patients (lower dose: *n* = 115; standard dose: *n* = 111).

Roxadustat Dose Adjustment Guidance

During the treatment period, the hemoglobin concentration was measured once every 2 weeks in the first 8 weeks, and then once every 4 weeks until week 16. Based on these measurements, the roxadustat doses were titrated to achieve and maintain a hemoglobin concentration of 100–120 g/L and to minimize the need for blood transfusion. It was recommended dose adjustments be made once every 4 weeks by considering both the current hemoglobin concentration and the change in the hemoglobin concentration over the previous 4 weeks. All physicians followed the same dose adjustment guidelines, as shown in **Supplementary Table S2**.

Supplemental Iron Use

Oral iron was not considered as rescue therapy and could be administered at any time according to clinical practice. Intravenous iron supplementation was only permitted as rescue therapy if, in the opinion of the investigator, the patient's hemoglobin had not responded adequately to oral iron; the patient could not tolerate oral iron; or the patient was iron-deficient (ferritin <100 ng/mL or transferrin saturation <20%). Once rescue therapy had been offered, the choice of intravenous iron therapy was at the discretion of the investigator and was administered according to the relevant approved package insert. The total dose of intravenous iron per treatment course was ≤1000 mg, and any single dose was required to be ≤400 mg. Ferritin and transferrin saturation were monitored every 4 weeks during the course of intravenous iron administration, and intravenous iron was discontinued when the patient was considered to no longer be iron-deficient (ferritin ≥100 ng/mL and transferrin saturation ≥20%). If the patient's hemoglobin had not responded adequately and ferritin or transferrin saturation remained at <100 ng/mL or <20%, respectively, at completion of the course of intravenous iron therapy, an additional course of intravenous iron was administered at the investigator's discretion. Ferritin and transferrin saturation

were reassessed within a minimum of 4–8 weeks following completion of the course of intravenous iron therapy.

Rescue Therapy

Rescue therapy guidelines were followed to standardize the use of rescue therapy and to ensure the safety of the individual patients. Rescue therapy included the administration of intravenous iron, blood transfusion, and erythropoiesis-stimulating agents. Rescue therapy was offered to patients whose medical condition warranted rescue therapy or who had hemoglobin <80 g/L, or a confirmed decrease of >10 g/L in hemoglobin from baseline and hemoglobin <90 g/L, with a confirmative test 5 days before initiating rescue therapy (unless it was urgent to take immediate action) to avoid local laboratory error.

Adverse Events of Special Interest

Post hoc analysis of adverse events of special interest was performed. These included cardiovascular events, venous thromboembolic events, serious infections, hyperkalemia, hypertension, seizures, secondary hypothyroidism, and severe cutaneous adverse reactions. These events were based on the important risks of roxadustat or important medical events of concern in previous clinical trials and post-marketing experience. The search strategies for the events above were as follows:

Cardiovascular events: The following four Standardized MedDRA Query (SMQ) Chinese search terms were used: “Haemorrhagic central nervous system vascular disorders (SMQ narrow),” “Ischemic central nervous system vascular disorders (SMQ narrow),” “Ischaemic heart disease (SMQ narrow),” and “Cardiac failure (SMQ narrow).”

Venous thromboembolism: The following SMQ search term was used: “Embolic and thrombotic events, venous (SMQ narrow).”

Serious infections: The following search term was used: “SOC Infections and infestations SAE."

Hyperkalemia: The following Preferred Term were used: “Hyperkalemia,” “Blood potassium increased,” and “Blood potassium abnormal.”

Hypertension: The following SMQ search term was used: “Hypertension (SMQ narrow).”

Seizures: The following SMQ search term was used: “Convulsions (SMQ narrow).”

Secondary hypothyroidism: The following SMQ search term was used: “Hypothyroidism (SMQ narrow).” The Preferred Term search terms were “Blood thyroid stimulating hormone decreased (PT)” and “Thyroxine free decreased (PT).”

Severe cutaneous adverse reaction: The following SMQ search term was used: “Severe cutaneous adverse reaction (SMQ narrow).”

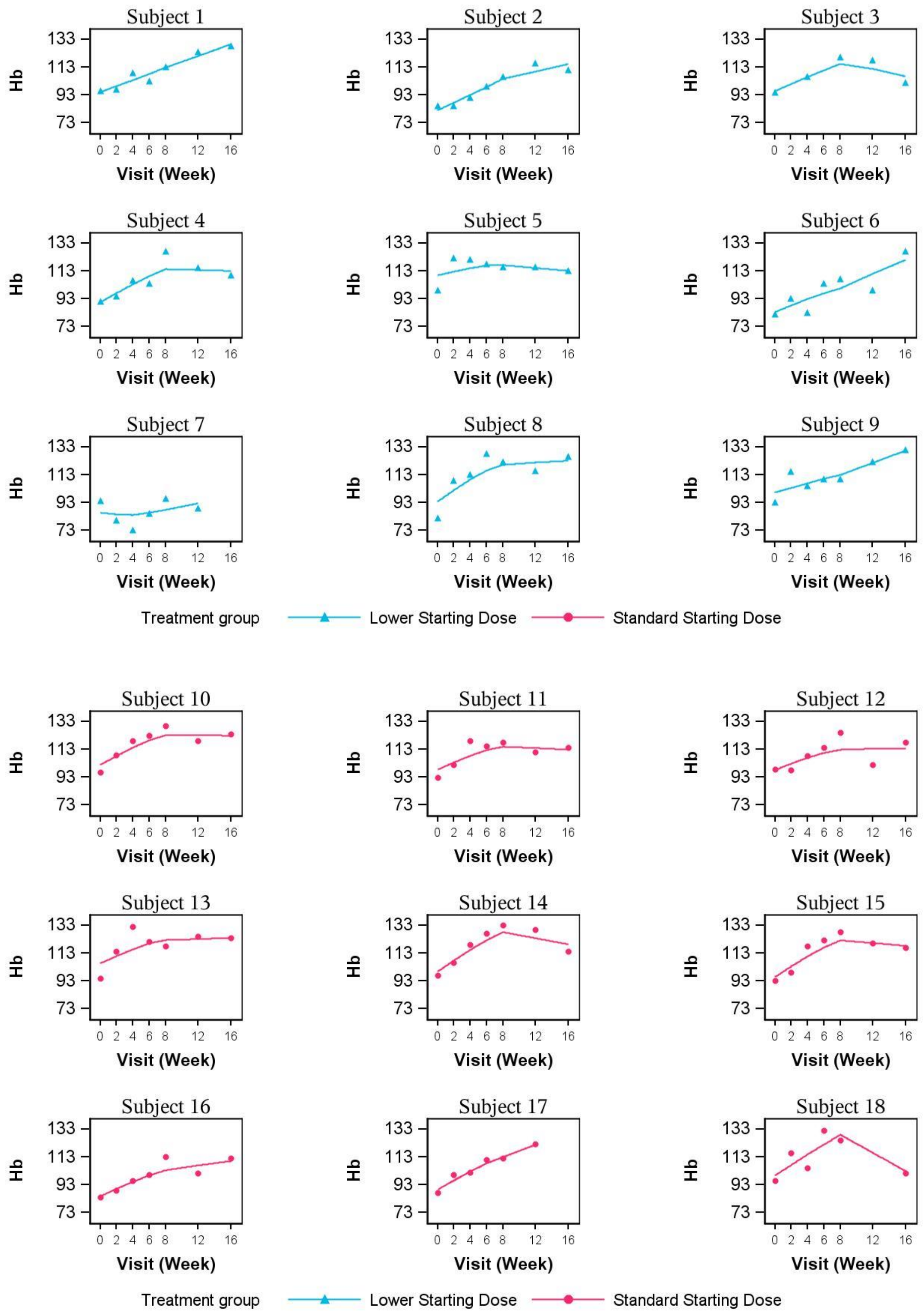

Supplementary Figure S1. Fitted trajectory of the Hb changes of the first nine patients by treatment group (full analysis set).

Hb, hemoglobin.

Supplementary Table S1. Additional inclusion and exclusion criteria

**Inclusion criteria**
Voluntary signed and dated informed consent approved by an ethics committee after the nature of the study had been explained and the patient had the opportunity to ask questions.
Agreement not to start taking any new traditional Chinese medicine for anemia and not to change the dose, schedule, or brand of any pre-screening traditional Chinese medicine for anemia from the beginning of the screening period through to the end of the trial.
**Exclusion criteria**
Anticipated renal replacement therapy within 6 months.
Life expectancy <12 months.
Blood transfusion within 12 weeks prior to Day 1 or anticipated need for transfusion.
Previous treatment with roxadustat or any HIF-prolyl-hydroxylase inhibitor within 12 weeks prior to Day 1.
Pregnancy or breastfeeding, or women of childbearing potential and men with sexual partners of childbearing potential not using adequate contraception.
New York Heart Association functional class III or IV congestive heart failure.
Myocardial infarction, acute coronary syndrome, stroke, seizure, or thromboembolic event (e.g., deep vein thrombosis or pulmonary embolism) within 26 weeks prior to Day 1.
Chronic inflammatory disease other than glomerulonephritis that could impact erythropoiesis (e.g., systemic lupus erythematosus, rheumatoid arthritis, and coeliac disease).
Known history of myelodysplastic syndrome, multiple myeloma, hereditary hematologic disease (e.g., thalassemia, sickle cell anemia, and pure red cell aplasia), other known causes of anemia other than chronic kidney disease, hemosiderosis, hemochromatosis, known coagulation disorder, or hypercoagulable condition.
Alanine aminotransferase or aspartate aminotransferase >3 × upper limit of normal or total bilirubin >1.5 × upper limit of normal at screening and baseline, except for patients with known Gilbert's syndrome.
Ferritin <100 ng/mL.
Positivity for human immunodeficiency virus antibody or hepatitis B surface antigen and hepatitis B virus-DNA level higher than the limits of detection, or positivity for hepatitis C virus antibody and hepatitis C virus-RNA higher than the limits of detection, or a scheduled anti-virus treatment for hepatitis B virus or HVC.
History of malignancy except cancers determined to be cured or in remission for ≥5 years, curatively resected basal cell or squamous cell skin cancers, or in situ cancer at any site.
Clinically significant active gastrointestinal bleeding (e.g., stool occult bleeding test positive for patient without taking oral iron or ≥++ for patients receiving oral iron).<br>Organ transplantation within 6 years or a scheduled organ transplantation.
Anticipated elective surgery that could lead to significant blood loss during the study period.
Deferoxamine, deferiprone, or deferasirox therapy within 12 weeks prior to Day 1 or anticipated use during the treatment period.
Unwilling to withhold intravenous iron during the treatment period.

Anabolic steroid treatment (e.g., androgen) within 12 weeks prior to Day 1 or anticipated use during the treatment period.
Use of an investigational medication or treatment, participation in an investigational interventional study within 28 days prior to day 1, or carryover effect of an investigational treatment expected during the study.
Any medical condition (e.g., active, clinically significant infection, decompensated cirrhosis) that in the opinion of the investigator may pose a safety risk to a patient in this study, may confound the efficacy or safety assessment, or may interfere with study participation.

Supplementary Table S2. Guidelines for roxadustat dose adjustment

**Change in hemoglobin over previous 4 weeks (g/L)**	**Hemoglobin concentration at the time of dose adjustment (g/L)**			
	**<105**	**105 to <120**	**120 to <130**	**≥130**
<−10	↑	↑	No change	Suspend dosing, monitor hemoglobin, reduce the dose by one step, and resume dosing when hemoglobin is <120 g/L
−10 to 10	↑	No change	↓	
>10	No change	↓	↓	

Notes for dose steps:

- Dose increases (↑) and reductions (↓) were preset according to the dose steps.
- The dose steps were as follows: 20 mg, 40 mg, 50 mg, 70 mg, 100 mg, 120 mg, 150 mg, and 200 mg three times per week.

For example, a dose of 70 mg would be increased to a dose of 100 mg. A dose of 150 mg would be reduced to 120 mg.

- The maximum dose was 2.5 mg/kg.

Dose adjustment for a rapid increase in hemoglobin concentration:

- If hemoglobin increased by >20 g/L over 2 weeks in a patient whose hemoglobin concentration was >90 g/L, the dose was reduced by one step.

Supplementary Table S3. Prior and concomitant oral and intravenous iron therapy (safety analysis set)

**Iron use**	**Lower dose** ***n*** **= 126**	**Standard dose** ***n*** **= 124**	**Overall** ***N*** **= 250**
**Prior oral iron use**	4 (3.2)	8 (6.5)	12 (4.8)
**Prior intravenous iron use**	14 (11.1)	15 (12.1)	29 (11.6)
**Concomitant oral iron use**	62 (49.2)	63 (50.8)	125 (50.0)
**Concomitant intravenous iron use**	5 (4.0)	2 (1.6)	7 (2.8)

Data are *n* (%).

Prior medications were defined as medications that ended prior to the first roxadustat dose.

Concomitant medications were defined as medications taken between the day of the first roxadustat dose and the day of the last roxadustat dose + 28 days, inclusive.

Supplementary Table S4. Sensitivity analysis of the mean change in hemoglobin from baseline averaged over weeks 12–16 (per-protocol set and full analysis set)

Visit	Statistic	Lower dose	Standard dose	Treatment difference [95% CI]
**Per-protocol set**				
Baseline[a]	*n*	115	111	
	Mean (SD)	89.44 (7.00)	90.59 (6.69)	
Average over weeks 12–16[b]	*n*	106	108	
	Mean (SD)	111.26 (13.38)	117.12 (9.97)	
Change from baseline in average over weeks 12–16 (MI ANCOVA)	LSM [95% CI]	21.47 [19.34, 23.60]	26.61 [24.52, 28.70]	−5.14 [−8.13, −2.15]
**Full analysis set**				
Baseline[a]	*n*	126	123	
	Mean (SD)	89.42 (6.96)	90.35 (6.83)	
Average over weeks 12–16[b]	*n*	115	119	
	Mean (SD)	110.42 (13.79)	116.21 (11.06)	
Change from baseline in average over weeks 12–16 (MI ANCOVA)	LSM [95% CI]	20.65 [18.46, 22.84]	25.85 [23.70, 28.00]	−5.20 [−8.27, −2.13]

CI, confidence interval; LSM, least-squares mean; MI ANCOVA, Multiple Imputation Analysis of Covariance Model; SD, standard deviation.

Supplementary Table S5. Proportion of patients achieving a hemoglobin response (full analysis set)

	**Lower dose** *n* **= 126**	**Standard dose** *n* **= 123**	**Odds ratio [95% CI]**[a]	***P* value**
Hemoglobin increase by ≥10 g/L, baseline to week 16[b]				
Cumulative patients, *n* (%)				
Baseline to week 8	108 (85.7)	115 (93.5)	0.415 [0.172, 0.999]	0.0498
Baseline to week 12	113 (89.7)	118 (95.9)	0.359 [0.123, 1.048]	0.0608
Baseline to week 16	115 (91.3)	118 (95.9)	0.436 [0.146, 1.302]	0.1370
	**Lower dose** *n* **= 126**	**Standard dose** *n* **= 123**	**Hazard ratio [95% CI]**	***P* value**
Time to first achieving hemoglobin increase of ≥10 g/L (days)[c]				
Patients censored (%)	11 (8.7)	5 (4.1)		
Median [95% CI]	29.0 [27.0, 29.0]	28.0 [17.0, 29.0]	0.756 [0.592, 0.965]	0.0250

CI, confidence interval.

[a]Comparison of proportions between the two groups was performed using the logistic regression model with the OR and its two-sided 95% CI.

[b]Baseline hemoglobin was defined as the average of least two values prior to the first dose.

[c]The time to first achievement of a hemoglobin increase of ≥10 g/L from randomization was analyzed using the Cox proportional-hazards ratio model as described for the time to rescue therapy, with baseline hemoglobin and baseline estimated glomerular filtration rate as covariates.

Supplementary Table S6. Roxadustat dose adjustment throughout the treatment period (full analysis set)

	**Lower dose** *n* **= 126**	**Standard dose** *n* **= 123**	**OR [95% CI]**[a]	***P* value**
Total number of dose increases	100	55	1.74 [1.25, 2.41]	0.0011
Dose increases per patient, mean (SD)	0.8 (0.92)	0.4 (0.73)		
Total number of dose reductions	125	138	0.90 [0.71, 1.15]	0.3885
Dose reductions per patient, mean (SD)	1.0 (0.78)	1.1 (0.98)		
Total number of dose suspensions	22	42	0.53 [0.32, 0.90]	0.0175
Dose suspensions per patient, mean (SD)	0.2 (0.40)	0.3 (0.51)		
Total number of dose adjustments	247	235	1.03 [0.86, 1.23]	0.7290
Dose adjustments per patient, mean (SD)	2.0 (0.90)	1.9 (0.98)		

CI, confidence interval; OR, odds ratio; SD, standard deviation.

[a]The Poisson regression model with robust error variance was used for treatment comparison including continuous baseline hemoglobin and baseline estimated glomerular filtration rate for adjustment.

Supplementary Table S7. Study drug exposure (safety analysis set)

	**Lower dose** ***n* = 126**	**Standard dose** ***n* = 124**	**Overall** ***N* = 250**
Actual exposure duration (weeks), mean (SD)	14.4 (3.5)	14.1 (3.7)	14.2 (3.6)
Total dose (mg), mean (SD)	2672.10 (1170.76)	3157.25 (1314.27)	2912.73 (1265.07)
Total cumulative dose (mg), median	2547.86	2929.29	2694.29
Average weekly dose (mg), mean (SD)	186.14 (64.71)	228.70 (75.82)	207.25 (73.46)
Treatment compliance (%), mean (SD)	98.65 (5.87)	98.85 (4.47)	98.75 (5.21)

Exposure duration = ((date of last dose − date of first dose) + 1) ÷ 7.

Study medication compliance was calculated as the number of doses administered during the period divided by the number of doses prescribed and expected to be taken during the same period (e.g., day 1 to the last dose) multiplied by 100.

SD, standard deviation.

Supplementary Table S8. Weekly roxadustat dose by visit (full analysis set)

Visit	Statistics	Lower dose (*n* = 126)	Standard dose (*n* = 123)	Overall (*N* = 249)
Weeks 1–2	*n*	126	123	249
	Mean (SD)	196.68 (33.25)	281.85 (49.88)	238.75 (60.01)
Weeks 3–4	*n*	123	121	244
	Mean (SD)	181.43 (37.74)	254.91 (50.0.)	217.87 (57.49)
Weeks 5–6	*n*	119	110	229
	Mean (SD)	198.39 (76.74)	246.41 (71.17)	221.46 (77.76)
Weeks 7–8	*n*	115	103	218
	Mean (SD)	187.79 (69.97)	232.77 (78.42)	209.04 (77.26)
Weeks 9–12	*n*	109	97	206
	Mean (SD)	183.74 (83.73)	213.02 (99.69)	197.53 (92.53)
Weeks 13–16	*n*	104	98	202
	Mean (SD)	181.55 (105.36)	192.90 (111.45)	187.06 (108.24)

SD, standard deviation.

Supplementary Table S9. Summary of AESI (safety analysis set)

**AESI Category**<br>**Preferred Term**	**Lower dose**<br>***n* = 126**	**Standard dose**<br>***n* = 124**	**Overall**<br>***N* = 250**
At least one AESI	42 (33.3)	30 (24.2)	72 (28.8)
Hyperkalemia	20 (15.9)	17 (13.7)	37 (14.8)
Hypertension	10 (7.9)	6 (4.8)	16 (6.4)
Hypertension	9 (7.1)	6 (4.8)	15 (6.0)
Blood pressure increased	1 (0.8)	0	1 (0.4)
Cardiovascular events	7 (5.6)	5 (4.0)	12 (4.8)
Cardiac failure	4 (3.2)	3 (2.4)	7 (2.8)
Acute coronary syndrome	1 (0.8)	0	1 (0.4)
Acute myocardial infarction	1 (0.8)	0	1 (0.4)
Arteriosclerosis coronary artery	1 (0.8)	0	1 (0.4)
Cardiac failure acute	1 (0.8)	0	1 (0.4)
Cardiac failure chronic	0	1 (0.8)	1 (0.4)
Cerebral infarction	0	1 (0.8)	1 (0.4)
Pulmonary oedema	1 (0.8)	0	1 (0.4)
Subclavian steal syndrome	1 (0.8)	0	1 (0.4)
Serious infections	9 (7.1)	2 (1.6)	11 (4.4)
Pneumonia	4 (3.2)	0	4 (1.6)
COVID-19	2 (1.6)	0	2 (0.8)
Biliary tract infection	0	1 (0.8)	1 (0.4)
Carbuncle	0	1 (0.8)	1 (0.4)
Chronic sinusitis	1 (0.8)	0	1 (0.4)
Herpes zoster	1 (0.8)	0	1 (0.4)
Pneumonia cryptococcal	1 (0.8)	0	1 (0.4)
Renal cyst infection	1 (0.8)	0	1 (0.4)
Venous thromboembolic events	0	0	0
Seizures	0	0	0

Secondary hypothyroidism	0	0	0
Severe cutaneous adverse reaction	0	0	0

Data are *n* (%).

AESI, adverse events of special interest.

MedDRA 25.1 was used for coding.

Patients were counted only once for each System Organ Class and Preferred Term.

Supplementary Table S10. Criteria for assessing major protocol deviations

Number	Major protocol deviation
1	Entry Deviation: Subject entered study, but did not satisfy eligibility criteria.
2	Withdrawal Deviation: Subject met withdrawal criteria during the study but was not withdrawn.
3	Investigational Product Deviation:
	A. Investigators prescribed wrong treatment or incorrect dose/dose adjustment, including incorrect timing of a dose.
	B. Subject received the wrong treatment or incorrect dose, including incorrect timing of a dose.
	a) Two or more consecutive missing doses or wrong doses in the first 8 weeks
	b) Incorrect starting dose
	c) Drug compliance <80% or >120%
4	Prohibited Medication Deviation: Subject received an excluded concomitant treatment
5	Significant noncompliance with study procedures that may impact the efficacy evaluation of the study drug will be evaluated case by case

Supplementary Table S11. Summary of serum iron parameters and changes from baseline by visit (full analysis set)

Item	Visit	Statistic	Lower dose *n* = 126	Standard dose *n* = 123	Overall *N* = 249
Iron (μmol/L)	Baseline	*n*	126	120	246
		Mean (SD)	13.23 (7.93)	13.03 (8.51)	13.134 (8.20)
	Week 8	*n*	110	102	212
		Mean (SD)	12.88 (5.77)	12.92 (6.25)	12.90 (6.00)
	Change from baseline at Week 8	*n*	110	102	212
		Mean (SD)	−0.58 (7.98)	−0.64 (11.11)	−0.61 (9.59)
	Week 16	*n*	96	93	189
		Mean (SD)	13.48 (5.498)	14.66 (6.33)	14.06 (5.94)
	Change from baseline at Week 16	*n*	96	91	187
		Mean (SD)	−0.22 (9.77)	1.58 (8.84)	0.66 (9.35)
Ferritin (μg/L)	Baseline	*n*	126	123	249
		Mean (SD)	346.85 (204.70)	386.86 (279.75)	366.61 (244.99)
	Week 8	*n*	110	102	212
		Mean (SD)	219.03 (235.69)	191.58 (216.48)	205.82 (226.53)
	Change from baseline at Week 8	*n*	110	102	212
		Mean (SD)	−121.53 (225.37)	−200.33 (232.21)	−159.44 (231.53)
	Week 16	*n*	96	93	189
		Mean (SD)	196.32 (218.14)	236.31 (268.30)	216.00 (244.29)
	Change from baseline at Week 16	*n*	96	93	189
		Mean (SD)	−158.90 (192.07)	−148.81 (165.73)	−153.93 (179.18)
Total iron-binding capacity (μmol/L)	Baseline	*n*	126	118	244
		Mean (SD)	44.37 (9.16)	42.23 (10.75)	43.33 (9.99)
	Week 8	*n*	110	102	212
		Mean (SD)	56.34 (12.84)	55.22 (12.02)	55.80 (12.43)
	Change from baseline at Week 8	*n*	110	100	210

Item	Visit	Statistic	**Lower dose** ***n* = 126**	**Standard dose** ***n* = 123**	**Overall** ***N* = 249**
		Mean (SD)	11.46 (12.32)	12.62 (12.80)	12.01 (12.54)
	Week 16	*n*	96	93	189
		Mean (SD)	53.76 (11.59)	54.50 (13.22)	54.12 (12.39)
	Change from baseline at Week 16	*n*	96	89	185
		Mean (SD)	8.75 (10.65)	11.79 (12.87)	10.21 (11.84)
Transferrin saturation (%)	Baseline	*n*	124	116	240
		Mean (SD)	29.26 (12.56)	31.22 (14.66)	30.21 (13.62)
	Week 8	*n*	110	102	212
		Mean (SD)	24.37 (13.14)	24.88 (13.90)	24.61 (13.48)
	Change from baseline at Week 8	*n*	108	99	207
		Mean (SD)	−4.81 (15.49)	−6.89 (20.01)	−5.80 (17.78)
	Week 16	*n*	96	93	189
		Mean (SD)	26.15 (11.45)	29.67 (16.53)	27.88 (14.25)
	Change from baseline at Week 16	*n*	94	88	182
		Mean (SD)	−3.28 (14.49)	−1.14 (19.31)	−2.24 (16.98)
Transferrin (g/L)	Baseline	*n*	126	119	245
		Mean (SD)	2.09 (0.40)	2.00 (0.36)	2.05 (0.38)
	Week 8	*n*	110	102	212
		Mean (SD)	2.61 (0.57)	2.57 (0.55)	2.59 (0.56)
	Change from baseline at Week 8	*n*	110	102	212
		Mean (SD)	0.49 (0.51)	0.55 (0.45)	0.52 (0.48)
	Week 16	*n*	96	93	189
		Mean (SD)	2.48 (0.53)	2.51 (0.55)	2.49 (0.54)
	Change from baseline at Week 16	*n*	96	91	187
		Mean (SD)	0.37 (0.47)	0.46 (0.45)	0.41 (0.46)

CONSORT 2010 checklist of information to include when reporting a randomised trial*

Section/Topic	Item No	Checklist item	Reported on page No
**Title and abstract**			
	1a	Identification as a randomised trial in the title	1
	1b	Structured summary of trial design, methods, results, and conclusions (for specific guidance see CONSORT for abstracts)	4
**Introduction**			
Background and objectives	2a	Scientific background and explanation of rationale	7–8
	2b	Specific objectives or hypotheses	8
**Methods**			
Trial design	3a	Description of trial design (such as parallel, factorial) including allocation ratio	8
	3b	Important changes to methods after trial commencement (such as eligibility criteria), with reasons	N/A
Participants	4a	Eligibility criteria for participants	8–9; Suppl. Table S1
	4b	Settings and locations where the data were collected	8
Interventions	5	The interventions for each group with sufficient details to allow replication, including how and when they were actually administered	9, Suppl. Methods
Outcomes	6a	Completely defined pre-specified primary and secondary outcome measures, including how and when they were assessed	10
	6b	Any changes to trial outcomes after the trial commenced, with reasons	N/A
Sample size	7a	How sample size was determined	10–11
	7b	When applicable, explanation of any interim analyses and stopping guidelines	N/A
Randomisation:			
Sequence generation	8a	Method used to generate the random allocation sequence	9
	8b	Type of randomisation; details of any restriction (such as blocking and block size)	9
Allocation concealment mechanism	9	Mechanism used to implement the random allocation sequence (such as sequentially numbered containers), describing any steps taken to conceal the sequence until interventions were assigned	9

Implementation	10	Who generated the random allocation sequence, who enrolled participants, and who assigned participants to interventions	9
Blinding	11a	If done, who was blinded after assignment to interventions (for example, participants, care providers, those assessing outcomes) and how	N/A
	11b	If relevant, description of the similarity of interventions	N/A
Statistical methods	12a	Statistical methods used to compare groups for primary and secondary outcomes	11
	12b	Methods for additional analyses, such as subgroup analyses and adjusted analyses	11
**Results**			
Participant flow (a diagram is strongly recommended)	13a	For each group, the numbers of participants who were randomly assigned, received intended treatment, and were analysed for the primary outcome	12, Suppl. Methods
	13b	For each group, losses and exclusions after randomisation, together with reasons	Fig. 1
Recruitment	14a	Dates defining the periods of recruitment and follow-up	8
	14b	Why the trial ended or was stopped	N/A
Baseline data	15	A table showing baseline demographic and clinical characteristics for each group	Table 1
Numbers analysed	16	For each group, number of participants (denominator) included in each analysis and whether the analysis was by original assigned groups	12–16
Outcomes and estimation	17a	For each primary and secondary outcome, results for each group, and the estimated effect size and its precision (such as 95% confidence interval)	12–15
	17b	For binary outcomes, presentation of both absolute and relative effect sizes is recommended	12–16
	18	Results of any other analyses performed, including subgroup analyses and adjusted analyses, distinguishing pre-specified from exploratory	15–16
Harms	19	All important harms or unintended effects in each group (for specific guidance see CONSORT for harms)	16
**Discussion**			
Limitations	20	Trial limitations, addressing sources of potential bias, imprecision, and, if relevant, multiplicity of analyses	19
Generalisability	21	Generalisability (external validity, applicability) of the trial findings	19
Interpretation	22	Interpretation consistent with results, balancing benefits and harms, and considering other relevant evidence	16–19
**Other information**			
Registration	23	Registration number and name of trial registry	3
Protocol	24	Where the full trial protocol can be accessed, if available	N/A
Funding	25	Sources of funding and other support (such as supply of drugs), role of funders	20

Citation: Schulz KF, Altman DG, Moher D, for the CONSORT Group. CONSORT 2010 Statement: updated guidelines for reporting parallel group randomised trials. BMC Medicine. 2010;8:18.

© 2010 Schulz et al. This is an Open Access article distributed under the terms of the Creative Commons Attribution License (http://creativecommons.org/licenses/by/2.0), which permits unrestricted use, distribution, and reproduction in any medium, provided the original work is properly cited.

*We strongly recommend reading this statement in conjunction with the CONSORT 2010 Explanation and Elaboration for important clarifications on all the items. If relevant, we also recommend reading CONSORT extensions for cluster randomised trials, non-inferiority and equivalence trials, non-pharmacological treatments, herbal interventions, and pragmatic trials. Additional extensions are forthcoming: for those and for up-to-date references relevant to this checklist, see www.consort-statement.org.
